# Patterns of Gene Flow in *Anopheles coluzzii* Populations From Two African Oceanic Islands

**DOI:** 10.1111/eva.70044

**Published:** 2024-11-26

**Authors:** Melina Campos, Gordana Rašić, João Viegas, Anthony J. Cornel, João Pinto, Gregory C. Lanzaro

**Affiliations:** ^1^ Vector Genetics Laboratory, Department of Pathology, Microbiology, and Immunology University of California—Davis Davis California USA; ^2^ Mosquito Genomics, QIMR Berghofer Medical Research Institute Herston Queensland Australia; ^3^ Centro Nacional de Endemias, Ministério da Saúde, Trabalho e Assuntos Sociais São Tomé Sao Tome and Principe; ^4^ Mosquito Control Research Laboratory, Department of Entomology and Nematology University of California Parlier California USA; ^5^ Global Health and Tropical Medicine, Instituto de Higiene e Medicina Tropical Universidade Nova de Lisboa Lisboa Portugal

**Keywords:** gene flow, genetically engineered mosquito, genomics, malaria, mosquito dispersal

## Abstract

The malaria vector *Anopheles coluzzii* is widespread across West Africa and is the sole vector species on the islands of São Tomé and Príncipe. Our interest in the population genetics of this species on these islands is part of an assessment of their suitability for a field trial involving the release of genetically engineered *A. coluzzii*. The engineered construct includes two genes that encode anti‐Plasmodium peptides, along with a Cas9‐based gene drive. We investigated gene flow among *A. coluzzii* subpopulations on each island to estimate dispersal rates between sites. Sampling covered the known range of *A. coluzzii* on both islands. Spatial autocorrelation suggests 7 km to be the likely extent of dispersal of this species, whereas estimates based on a convolutional neural network were roughly 3 km. This difference highlights the complexity of dispersal dynamics and the value of using multiple approaches. Our analysis also revealed weak heterogeneity among populations within each island but did identify areas weakly resistant or permissive of gene flow. Overall, *A. coluzzii* on each of the two islands exist as single Mendelian populations. We expect that a gene construct that includes a low‐threshold gene drive and has minimal fitness impact should, once introduced, spread relatively unimpeded across each island.

## 1 Introduction

Malaria is an ancient disease with references dating back to Chinese documents as early as 2700 BC (Cox 2010). This parasitic disease is caused by protozoans of the genus *Plasmodium* and is transmitted to humans by the bite of infected *Anopheles* mosquitoes. Africa bears the largest burden of malaria globally, where, in the year 2021, 96% of the estimated 619,000 malaria deaths occurred (WHO 2022). The most widely used and effective malaria control methods focus on vector control, specifically the use of insecticide‐treated nets (ITNs) and the application of indoor residual spraying (IRS). However, these approaches have limitations due to logistical challenges associated with their application, cost, and human non‐compliance (Monroe et al. 2021). In addition, the emergence of insecticide resistance poses a significant threat to their efficacy (Hamel et al. 2011). In response to these challenges, new technologies, such as those involving genetically engineered mosquitoes (GEM) with gene‐drive, are under development and could potentially offer alternatives to traditional vector control methods (Carballar‐Lejarazú et al. 2020; Hammond et al. 2016; Hoermann et al. 2022; Kyrou et al. 2018). Gene‐drive has not, to date, been evaluated in a field setting, but field trials are urgently needed to advance this promising technology. Considering both existing and emerging malaria vector control strategies, comprehensive information on the vector's spatial dispersal and population structure remains crucial (Godfray 2013).

Oceanic islands have been considered as among the most suitable sites for initial field trials of GEMs due to their geographic isolation, small size, and hard boundaries (James et al. 2018; Lanzaro et al. 2021). The island nation of São Tomé and Príncipe (STP) has been selected for study as a potential GEM field trial site (Lanzaro et al. 2021). Located in the Gulf of Guinea off the coast of West Africa, STP hosts a single malaria vector species, *Anopheles coluzzii*. Colonization of STP by *A. coluzzii* occurred about 500 years ago, coinciding with human colonization of the islands (Ditter et al. 2022). Studies of the population genetics of *A. coluzzii* in STP have provided evidence for highly restricted migration between populations from these islands and those in mainland West Africa (Campos et al. 2021; Marshall et al. 2008; Salgueiro et al. 2013).

Here, we investigate *A. coluzzii* populations in São Tomé and Príncipe, with a focus on describing within‐island population structure and understanding spatial dispersal patterns. This study offers an opportunity to explore gene flow in an environment with discreet boundaries imposed by the Atlantic Ocean and across two island settings that dramatically differ with respect to size (total area of São Tomé = 854 km^2^; Príncipe = 142 km^2^). In addition, both islands are of volcanic origin with associated topographic discontinuities that offer opportunities to identify gene flow barriers and corridors. From a practical perspective, this study informs the logistics of any proposed release of GEMs into this environment and contributes to the development of mathematical models to predict/describe their pre‐ and post‐release dynamics.

## 2 Materials and Methods

### 2.1 Field Collections

**FIGURE 1:**
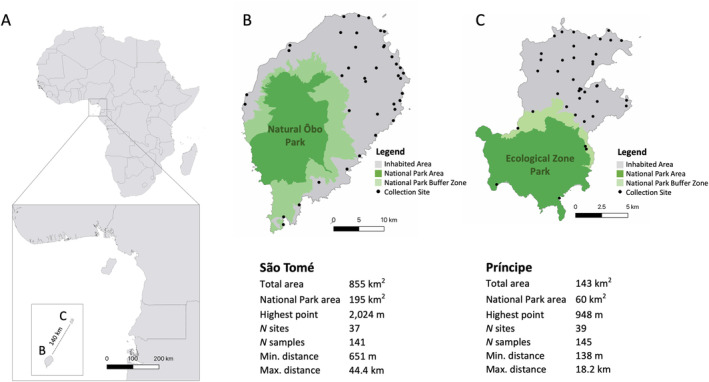
Sampling locations and geographic features. (A) São Tomé and Príncipe (STP) is a nation comprised of these two islands, São Tomé and Príncipe (within the square), located in the Gulf of Guinea off the coast of central Africa. *Anopheles coluzzii* specimens were collected from a total of 37 sites in São Tomé (B) and 39 sites in Príncipe (C). Collection sites are represented by black dots on the maps. São Tomé Island is about six times larger in area than Príncipe. The national parks (indicated in dark green) cover approximately 22% of São Tomé and 42% of Príncipe. “Min. and Max. distance” in the tables beneath each map correspond to the minimum and maximum distances between pairs of collection sites.

Field collections of immature stages of *Anopheles coluzzii* were conducted in December 2019 on the São Tomé and Príncipe islands. Specimens were collected from various aquatic larval breeding sites, including temporary sources such as roadside pools and shallow puddles between houses and semi‐permanent sites such as ponds. The sample sites covered the geographical space occupied by villages and hamlets, which represents a majority of the total inhabited area on each island, and the environment utilized by this synanthropic mosquito species. Sites were in 39 localities in Príncipe and 37 in São Tomé (Figure 1). In addition to the collections made in 2019, seven samples from remote sites in Príncipe were obtained in March 2022 and were added to expand the spatial representation of the *A. coluzzii* population on this island.

After morphological identification, specimens were transferred to individual labeled tubes containing 80% ethanol for preservation until further processing in the laboratory. Geographic coordinates for each survey site were obtained using a Garmin GPSMAP 64SC, and maps were created using the QGIS application (accessible at: http://qgis.osgeo.org).

### 2.2 Sample Processing

Genomic DNA was extracted from individual mosquito samples using a Biosprint machine (Qiagen, Hilden, Germany), following established protocols (Nieman et al. 2015). Each specimen was further identified as *A. coluzzii* using the molecular diagnostic protocol known as the Divergence Island SNPs (DIS) assay (Lee et al. 2014). DNA yield was measured using a dsDNA high‐sensitivity assay kit on a Qubit instrument (Thermo Fisher Scientific, Waltham, MA, USA). Only females were selected for DNA library preparation. Males were identified and excluded based on a positive test for the presence of a specific Y‐chromosome region using S23 primers described by Krzywinski et al. (2004). Individual genomic DNA libraries were prepared using the KAPA HyperPlus Kit (Roche Sequencing Solutions, Indianapolis, IN, USA), with 10 ng of input DNA, as previously described (Yamasaki et al. 2016). Libraries were then size selected and cleaned up using AMPure SPRI beads (Beckman Coulter Life Sciences, Indianapolis, IN, USA). Individual libraries were equimolarly pooled and sequenced using an Illumina HiSeq 4000 instrument (Illumina, San Diego, CA, USA) at the UC Davis DNA Technologies Core facility.

### 2.3 Whole Genome Sequencing

Raw Illumina reads were first demultiplexed and then filtered and trimmed using Trimmomatic v0.36 (Bolger, Lohse, and Usadel 2014). Clean reads were mapped to the reference genome assembly AgamP4 (Holt et al. 2002; Sharakhova et al. 2007) using BWA‐MEM v0.7.15 (Li 2013) with default settings. PCR duplicate reads were removed using Sambamba markdup (Tarasov et al. 2015). Freebayes v1.2.0 (Garrison and Marth 2012) was used for variant calling (“standard‐filters,” “‐no‐population‐priors,” “theta = 0.01,” and “max‐complex‐gap = 0”). The resulting set of variants were normalized with vt normalize v0.5 (Tan, Abecasis, and Kang 2015), and those lacking support from both overlapping forward and reverse reads were filtered out using vcffilter v1.0.0rc2 (https://github.com/vcflib/vcflib). Only biallelic SNPs with a minimum depth of 8 and a minimum quality of 20 were retained for further analysis.

### 2.4 Population Genetic Structure and Diversity

Population structure analysis was performed in two ways: first, using the combined dataset from both São Tomé and Príncipe, and second, using separate datasets for each island. For these analyses, we removed any SNP that had > 10% missingness, minor allele frequency (MAF) < 5%, and was located in a heterochromatic region of the genome (Sharakhova et al. 2010). Species in the *A. gambiae* complex are known to commonly carry several paracentric chromosome inversions on chromosome 2 (Coluzzi et al. 2002), which may confound population genomics studies. However, *A. coluzzii* populations in São Tomé and Príncipe are monomorphic for the standard chromosome 2 arrangements (Pinto et al. 2000), corresponding to the FOREST chromosomal form (Coluzzi, Petrarca, and Di Deco 1985). Therefore, SNPs on chromosome 2 were not filtered out in our study. All analyses were performed on the autosomes, except for the whole genome scan analysis.

Principal Component Analysis (PCA) was performed after pruning for significant linkage disequilibrium (LD) using *SNPRelate* v3.1.3 (Zheng et al. 2012) and *adegenet* v2.1.4 (Jombart et al. 2017) packages in R (R Core Team 2021). Within‐island analysis included PCA, with resulting 2D plots color‐coded based on k‐means clustering of geographic location of the samples. *K*‐means clustering was performed in R, with a fixed number of 12 clusters for each island dataset. Outliers in the 2D plots were defined based on the mean and standard deviation of the first two principal components (PCs), using a threshold of three standard deviations. Ancestry components were assigned to each individual using Bayesian analysis implemented in ADMIXTURE v1.3.0 (Alexander, Novembre, and Lange 2009). For this analysis, we randomly sampled three independent replicates of 50,000 SNPs from the filtered dataset. Each replicate underwent 10 iterations for values of K clusters ranging from 1 to 10. The best‐fitting *K* was determined based on the lowest cross‐validation error values. The results were compiled using the online version of CLUMPAK (Kopelman et al. 2015).

To assess overall genetic differentiation between populations on the two islands and sites within each island, we calculated Hudson's fixation index (*F*_ST_) using *scikit‐allel* v 1.2.0 (Alistair and Harding 2017; Hudson, Slatkin, and Maddison 1992). For the within‐island analysis, we included collection sites with a sample size (*N*) of four or more. A neighbor‐joining tree was constructed based on pairwise *F*_ST_ between collection sites within each island using the *ape* package in R. Nucleotide diversity (π) and Tajima's *D* were calculated in non‐overlapping windows of 10 kb on euchromatic regions of the genome using VCFtools (Danecek et al. 2011). To investigate inbreeding, run of homozygosity (ROH) analysis was performed in PLINK (Chang et al. 2015). For the whole genome scan analysis, we calculated the *F*_ST_ in non‐overlapping windows of 10 kb between populations from the two islands using VCFtools (Danecek et al. 2011). All resulting plots were generated using *ggplot2* (Wickham 2016) in R (R Core Team 2021).

### 2.5 Genetic and Geographic Distance Matrices

To investigate the correlation between genetic and geographic distance within each island, we generated individual‐based distance matrices. A dissimilarity matrix with average pairwise difference in PLINK was calculated for genetic distances (Chang et al. 2015). For geographic distances, we calculated the great‐circle distance between collection sites in two dimensions based on their latitude and longitude coordinates, considering Earth's curved surface as 6371 km.

### 2.6 Estimation of Effective Dispersal

#### 2.6.1 Isolation‐by‐Distance (IBD)

A Mantel's test, implemented in *ecodist* (Goslee and Urban 2007) in R, was used to assess IBD among individuals within each island. The analyses excluded closely related individuals to ensure that it reflected the correlation between genetic and geographic distances without being confounded by the inclusion of kinship pairs. We measured a relationship coefficient using the *relatedness2* function in VCFtools (Danecek et al. 2011) and applied a cutoff of 0.15 (Manichaikul et al. 2010).

#### 2.6.2 Spatial Autocorrelation

We estimated mosquito effective dispersal range using a spatial autocorrelation analysis with a Mantel correlogram. Assessment of the degree of similarity between spatial and genetic distances was performed over 1 km intervals using a multivariate autocorrelation function from the *ecodist* package (Goslee and Urban 2007) in R (R Core Team 2021). The autocorrelation coefficient was generated by 100 permutations of individuals among their geographic locations. For the spatial autocorrelation, matrices from both São Tomé and Príncipe islands were combined to obtain the overall dispersal capacity of *A. coluzzii*. A positive correlation coefficient (Mantel *r*) indicates that genetically similar individuals are more likely to occur close to each other in space. Conversely, negative values suggest an inverse relationship, while values close to zero indicate no spatial pattern.

#### 2.6.3 Dispersal Distance Using a Convolutional Neural Network

We also applied deep learning to estimate the mean per‐generation dispersal distance using *disperseNN2* (Smith and Kern 2023). This tool uses SNP data in standard variant call format (VCF) and geographic coordinates for each sample to build a pairwise convolutional network. The program includes simulations of a training dataset designed to closely resemble the empirical data. Our empirical datasets consisted of latitude and longitude metadata along with 10,000 biallelic SNPs for each island. The parameters used were: expected dispersal distance for *A. coluzzii* ranging from 0.5 to 6.5 km, maximum generations of 1000, species width range of 50 km, and population density of five individuals per km^2^. We used the script available from the *disperseNN2* repository (https://github.com/kr‐colab/disperseNN2) to perform the simulation step using SLiM v 4.0.1 (Haller and Messer 2019). A post hoc correction was applied to the dispersal output, as recommended in Smith et al. (2023).

#### 2.6.4 Estimated Effective Migration Surfaces

We applied the Estimated Effective Migration Surfaces (EEMS) analysis to visualize rates of gene flow within the geographical space of each island (Petkova, Novembre, and Stephens 2016). EEMS employs a systematic grid to model the relationship between genetics and geography, identifying areas where effective migration deviates from expectations—either lower than expected (indicating resistance), higher than expected (indicating facilitation) or exhibiting a neutral impact. For this analysis, three components were used: (1) a genetic dissimilarity matrix, as described above, (2) a polygon defining the geographic area to be included, and (3) the coordinates for each sample. The EEMS analysis was conducted in three independent runs using the Markov Chain Monte Carlo (MCMC) algorithm, and results were averaged. Each MCMC run consisted of 10 million burn‐in iterations and 10 million post‐burn‐in iterations thinned by an interval of 5000 iterations. The number of demes corresponded to the number of collection sites on each island. The output from this analysis was processed using the R package EEMSplots (Petkova, Novembre, and Stephens 2016).

### 2.7 Statistics and Reproducibility

A total of 286 specimens of *A. coluzzii* collected across São Tomé and Príncipe Islands were used for this study. Statistical analyses were conducted using R, and the corresponding *p* values are presented in the text and/or figures.

## 3 Results

We sequenced the whole genomes of a total of 286 field collected female *A. coluzzii*, 141 from São Tomé and 145 from Príncipe (Figure 1). Samples were distributed across 37 sites in São Tomé, with *N* ranging from 2 to 5 individuals per site, and 39 sites in Príncipe, with *N* per site ranging from 1 to 6. Sequencing generated a combined total of approximately 9 billion reads. On average, each sample had genome coverage of approximately 14× (Table S1).

### 3.1 Between‐Island Analysis

**FIGURE 2:**
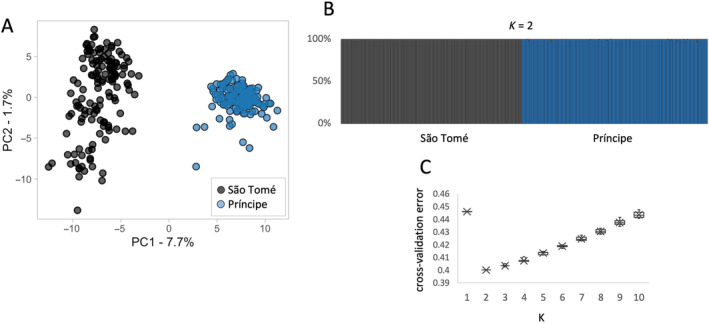
Population structure by principal component (PCA) and admixture analyses. (A) Plot of the first two components of PCA. (B) Individual ancestry estimation with ADMIXTURE. This Bayesian analysis was based on 10 independent replicates for each K. (C) Cross‐validation error for K from 1 to 10 of ADMIXTURE analysis; the lowest value is the best‐fit number of clusters. Analyses were based on 50,000 SNPs on the autosomal genome.

We initiated our investigation into the genetic structure of *A. coluzzii* populations with a comparison of populations from São Tomé with those from Príncipe. Results were consistent with earlier reports of considerable genetic differentiation between populations on the two islands (Campos et al. 2021; Ditter et al. 2022). We performed dimensionality reduction analysis on the SNP dataset using a linear transformation by PCA. Differentiation between island populations was evident in the output, with two distinct and well‐defined clusters identified in the 2D plots (Figure 2A,B). An ADMIXTURE analysis confirmed the distinction between São Tomé and Príncipe populations, with the optimal number of genetic clusters being 2, one representing each island (Figure 2C,D). The overall *F*_ST_ value between São Tomé and Príncipe populations was 0.114. This value for *F*_ST_ is consistent with estimates among *A. coluzzii* populations in mainland Africa (Campos et al. 2021; Miles et al. 2017). The *F*_ST_ genome scan analysis revealed that genetic differentiation is distributed across the genome, with the X chromosome showing the highest values (Figure S1).

**FIGURE 3:**
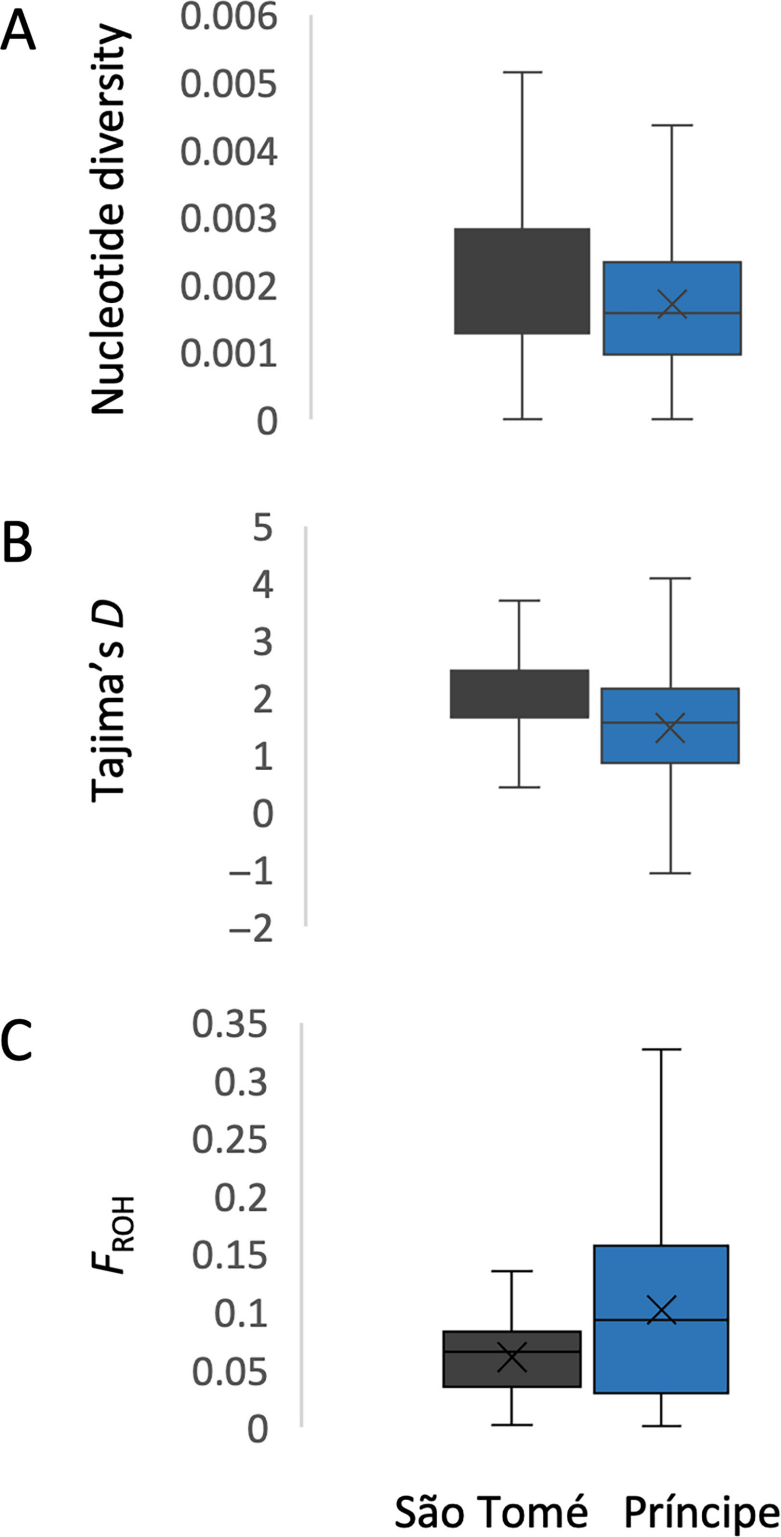
Population genetic metrics. Boxplots of metrics describing populations of *Anopheles coluzzii* from São Tomé and Príncipe. (A) Nucleotide diversity in 10 kb windows. (B) Tajima's *D* in 10 kb windows. (C) Frequency of runs of homozygosity (FROH). All analyses were performed on autosomes. For all boxplots, outlier points are not included.

Mean nucleotide diversity was higher in São Tomé (mean: 1.65 × 10^−3^) compared to Príncipe (mean: 1.33 × 10^−3^); however, both were lower than levels found in mainland populations (Campos et al. 2021) (Figure 3A). Tajima's *D* values throughout the genome were mostly positive for both island populations (mean value for São Tomé: 2.02; mean value for Príncipe: 1.46), but Príncipe presented a wider variation (Figure 3B). The genomes of individuals from São Tomé contained ~6% stretches, or runs, of sequential homozygous genotypes (ROH), while individuals from Príncipe had as high as 10% ROH (Figure 3C).

### 3.2 Within‐Island Analysis

**FIGURE 4:**
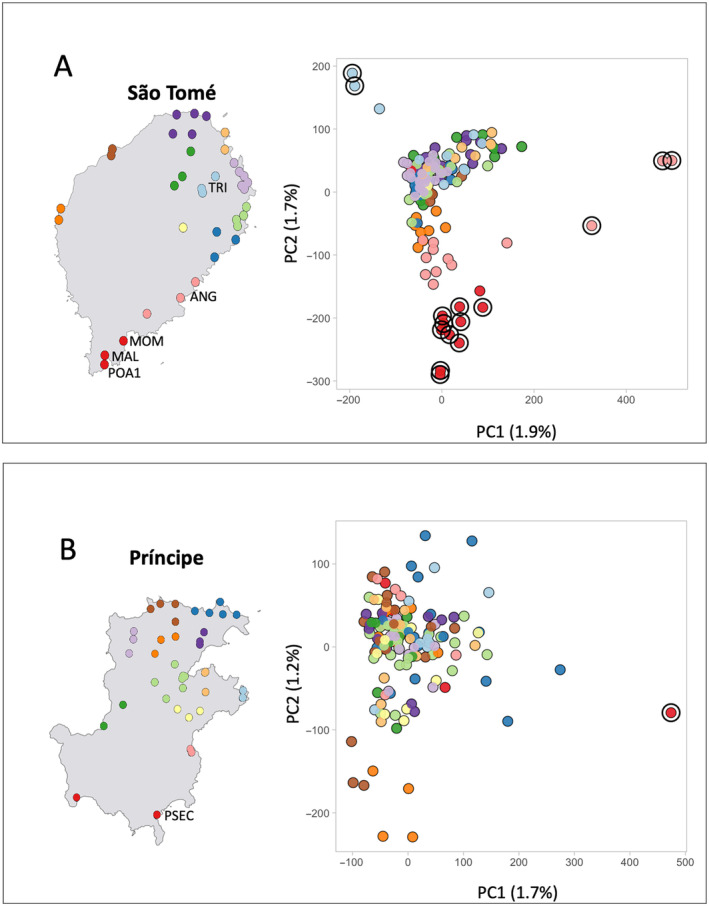
Within‐island population structure. Collection sites were grouped and colored based on *k*‐means clustering analysis of geographic coordinates (*K* = 12) in São Tomé (A) and Príncipe (B). PCA results are shown for both islands. Black circles highlight outliers, and the three‐letter codes on the map indicate the origin of these outliers.

To facilitate visualization in the PCA, collection sites were grouped by geographic location using k‐means analysis in the 2D‐plots (Figure 4 and Figure S2). Overall, variance between sites within islands was low. The first and second principal components each explain less than 2% of the total variance. PCA revealed weak groupings of *A. coluzzii* populations in São Tomé, with those populations in the southernmost part of the island (Figure 4A) being distinct. Of the total 25 samples from six collection sites in the south (pink and red dots in Figure 4A), thirteen were outliers: three from Angolares (ANG), four from Malanza (MAL), two from Monte Mario (MOM) and four from Porto Alegre (POA1). The remaining outliers from other regions were all from Trindade (TRI) in the east‐central part of the island (Figure 4A). In contrast, PCA analysis performed with the samples from Príncipe Island showed no detectable signal of population structure (Figure 4B). There was one outlier among the six samples from Praia Seca (PSEC), located in the southernmost region of the island (Figure 4B). Praia Seca is a temporary fishing village which is uninhabited most of the year, suggesting that this population experiences regular crashes and possibly repeated extinction and reestablishment.

Pairwise *F*_ST_ values between sites on São Tomé Island were generally low (overall average *F*_ST_ = 0.014). However, higher values (average *F*_ST_ = 0.027) were observed between the southern sites and the remaining sites in São Tomé (Table S2). For example, the southern collection sites of Malanza (MAL) and Angolares (ANG) exhibited the highest average *F*_ST_ values (0.053 and 0.052, respectively) (Figure S3A and Table S2). These represent moderate levels of genetic divergence (Balloux and Lugon‐Moulin 2002; Hartl 1997).

In Príncipe, the average *F*_ST_ value between all collection sites was an order of magnitude lower than São Tomé (average *F*_ST_ = 0.007), suggesting very little differentiation among sites on the island (Figure S3B and Table S3). Higher values were observed among a few scattered coastal collection sites, for example, Bombom (BOM) and Ribeira Ize (REZ) in the extreme north (average *F*_ST_ = 0.032 and 0.028).

**FIGURE 5:**
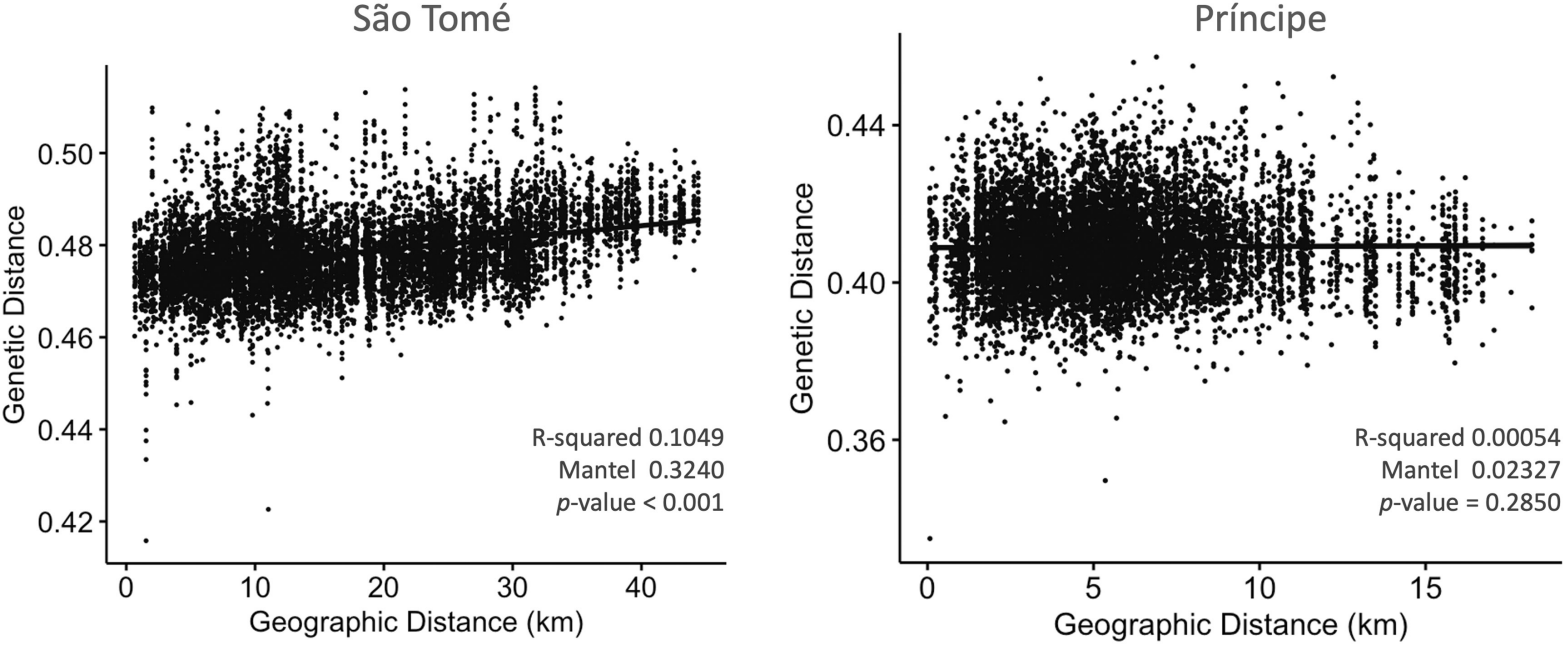
Isolation‐by‐distance analysis. Mantel test and linear regression were performed on the matrices of genetic dissimilarity and linear geographic distance (km) between sample pairs in São Tomé and Príncipe. First degree close kin individuals were removed from both datasets.

**FIGURE 6:**
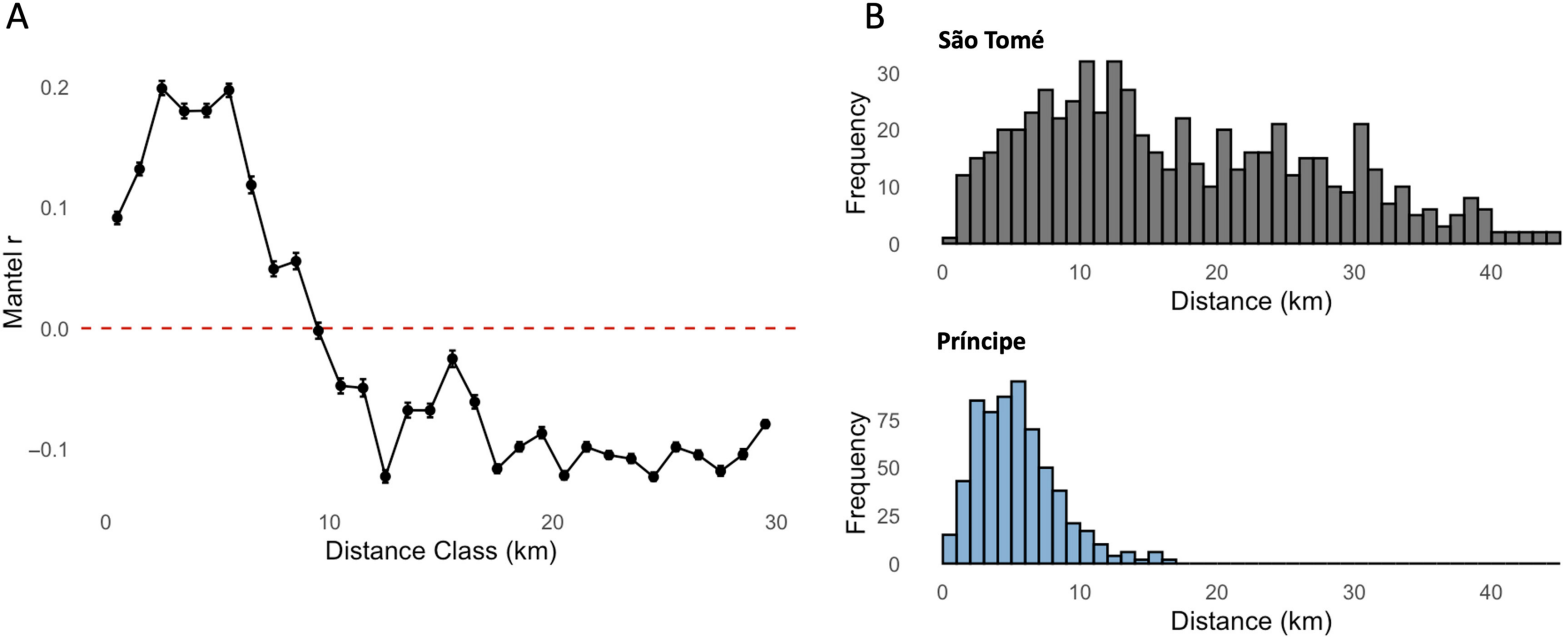
Spatial autocorrelation. (A) Mantel correlograms in 1 km intervals for combined matrices of genetic and geographic distances of São Tomé and Príncipe. The y‐axis shows the Mantel r coefficient for each distance in kilometers on the x‐axis. Vertical lines represent the lower and upper bounds of the confidence limit for Mantel *r*. (B) Histogram the distribution of distances between collection sites in São Tomé (top) and Príncipe (bottom).

A statistically significant but weak positive correlation (*R* ^2^ = 0.1049, *p*‐value < 0.001) was found between genetic distance (genetic dissimilarity) and linear geographic distance among individuals sampled from São Tomé (Figure 5). There was no correlation between geographic and genetic distance found in Príncipe (*R* ^2^ = 0.0005, *p*‐value = 0.285; Figure 5). Spatial autocorrelation analysis revealed a significant positive correlation (i.e., high genetic similarity) among individuals at distances up to ~7 km, with the highest correlation coefficient at kilometers 3–6 (Figure 6A). São Tomé is a larger island, with a maximum distance between collection sites of 44.4 km, whereas the most distant sites sampled in Príncipe were 18.2 km apart (Figure 6B). The average geographic distance between collection sites on São Tomé was 17.0 km, while on Príncipe, it was 5.8 km. In Príncipe, approximately 72% of the collection sites fell within the positive spatial autocorrelation range, suggesting that the entire island lies within the effective dispersal range of *A. coluzzii* and that on this island it exists as a single Mendelian population.

In addition, we employed a convolutional neural network (CNN) to estimate the per‐generation dispersal distance of *A. coluzzii*. For this analysis, we conducted training, validation and empirical runs for each island. The training and validation phases demonstrated that the model fit the dataset reasonably well, as shown in Figures S4 and S5. These figures indicate that the model was able to capture the key dispersal dynamics of the populations, ensuring reliable extrapolation to the empirical data. Our empirical analysis revealed similar dispersal estimates for both the São Tomé and Príncipe islands. For São Tomé, the mean per‐generation dispersal distance was 2.58 km (SE = 0.05), while for Príncipe, it was slightly higher at 2.83 km (SE = 0.09). The low standard errors associated with these estimates indicate a high level of precision in our results.

**FIGURE 7:**
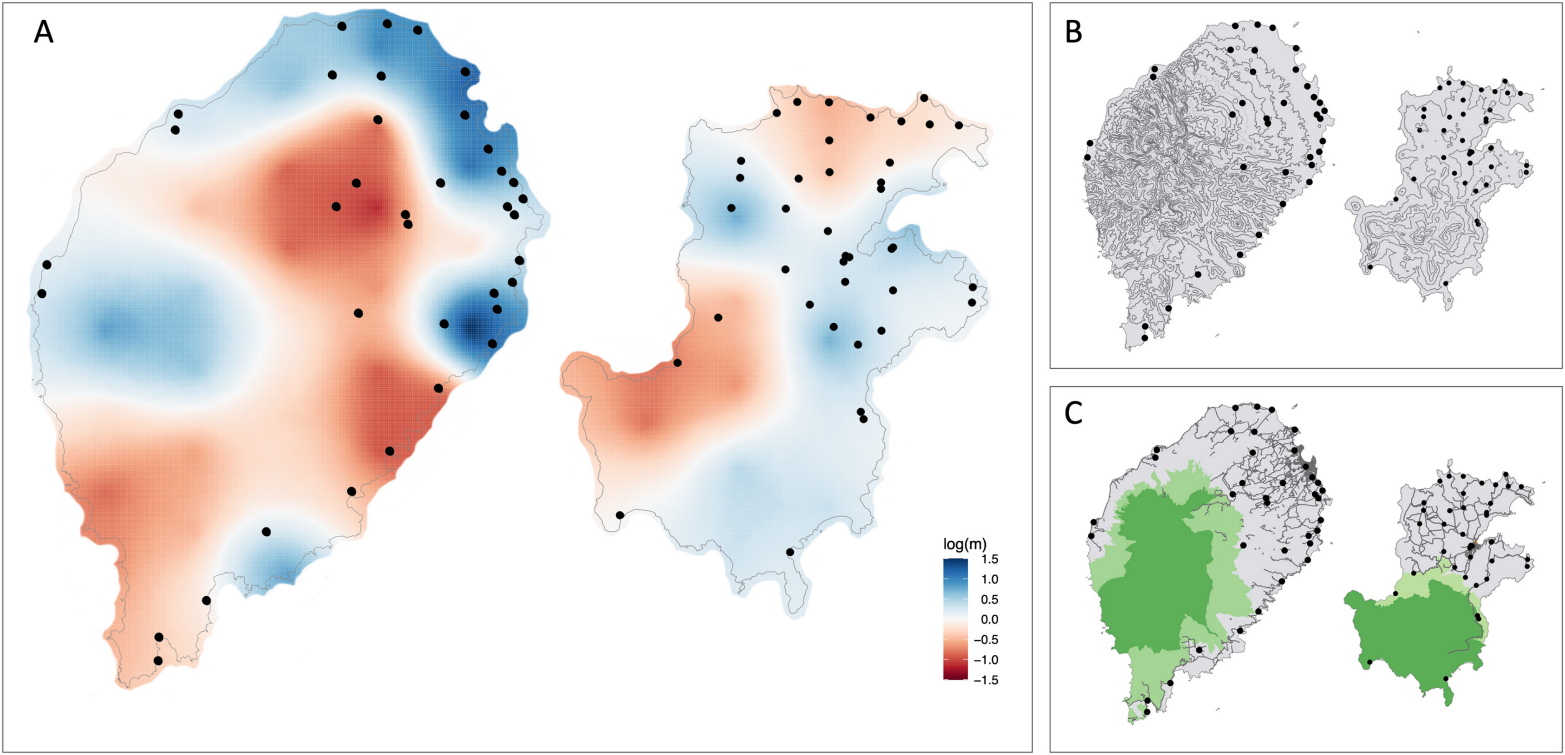
Estimated Effective Migration Surfaces (EEMS) for *Anopheles coluzzii* populations in São Tomé and Príncipe. (A) Maps overlaid with a color gradient representing the log of effective migration rates (*m*) estimated by EEMS for São Tomé and Príncipe. Rates higher than the overall average *m* are depicted in blue, while rates lower than the overall average are depicted in dark orange. (B) Map of São Tomé and Príncipe with elevation contour information at 100‐m intervals. (C) Map of São Tomé and Príncipe with roads and National Parks layers. All maps are not scaled and black dots represent collection sites.

Estimated Effective Migration Surfaces analysis revealed distinctions in the patterns of gene flow among *A. coluzzii* populations in São Tomé relative to Príncipe. Populations in São Tomé displayed a wider range of effective migration rates compared to those in Príncipe (Figure 7). More specifically, in São Tomé, EEMS revealed three distinct areas where migration rates were lower than expected, indicating areas where resistance to gene flow was high (Figure 7A). Two of these areas are in the southern half of the island, while the third is in the north‐central region, an area of higher elevation (Figure 7A,B). Conversely, northeastern São Tomé displayed higher than expected effective migration rates between *A. coluzzii* populations (Figure 7A); a higher density of roads and people in this part of the island may facilitate dispersal (Figure 7C). In Príncipe, effective migration rates were generally high across the island, except for two areas of slight resistance to gene flow, one in the northern and another in the western region (Figure 7A).

## 4 Discussion

Dispersal and associated gene flow are central forces driving organic evolution. For species that reside on isolated islands, dispersal is constrained by the dire consequences faced by individuals that move beyond the coastline into the open ocean. This constraint may represent a powerful force for selection favoring reduced capacity for dispersal (Whittaker and Fernández‐Palacios 2007). Species assemblages residing in island archipelagos have been central to our understanding of evolution going back to Darwin (1859). The major body of work at that time, and since, has focused on the evolutionary consequences of limited dispersal between island populations or between these and mainland populations. Our focus here is within‐island dispersal among local populations of the mosquito species *Anopheles coluzzii*, a major malaria vector in western Africa. Extensive investigations of dispersal among malaria vectors are driven by its direct impact on disease transmission and the development of effective control strategies.

In general, mosquitoes are thought to be poor flyers (Bomphrey et al. 2017), but recent work suggests that, in some situations, wind‐assisted long‐distance dispersal can occur (Atieli et al. 2023). Previous studies have demonstrated that the genetics of *A. coluzzii* populations from São Tomé and Príncipe correspond with their geographic isolation and that immigration to and from the islands was not detected (Campos et al. 2021; Lanzaro et al. 2021; Pinto et al. 2002). In the present study, we found moderate to high levels of genetic differentiation (*F*_ST_) between *A. coluzzii* populations from these two islands, corroborating earlier studies (Campos et al. 2021). Furthermore, both island populations exhibit positive Tajima's *D* values, indicating a strong deficit of rare alleles. This pattern is typically observed in small, isolated populations, such as those on remote oceanic islands like STP, which often experience bottlenecks (Marshall et al. 2008; Tajima 1989). Notably, a previous study revealed that the effective population size trajectory for both islands showed a sharp decrease, indicative of a founder effect, suggesting that only a small fraction of the ancestral population from mainland Africa successfully established itself on the islands (Campos et al. 2021).

Diverging patterns between the island populations were observed in terms of genetic diversity and run of homozygosity (ROH) analysis. São Tomé displayed higher genetic diversity and shorter stretches of homozygosity compared with populations on Príncipe. As a comparison, homozygosity in the Príncipe population was lower than what is typically observed in long‐standing laboratory colonies of *A. coluzzii*, where high inbreeding is expected (Miles et al. 2017). This is in agreement with a more recent colonization of Príncipe island, possibly from rare historical episodes of immigration from the main island of São Tomé with no evidence for immigration from the large continental populations (Ditter et al. 2022).

Through whole genome sequencing of samples collected from numerous locations across both islands, we obtained a finer‐scale resolution of the genetic structure and gene flow patterns in *A. coluzzii* populations. Studies conducted on mainland Africa using mark‐release‐recapture (MRR) experiments have reported the capacity of *Anopheles* mosquitoes to travel average distances of about 1041 m per day, as reviewed by Verdonschot and Besse‐Lototskaya (2014). However, dispersal range can vary depending on seasonal, topographic and ecological factors, and MRR studies often operate on a scale that may underestimate maximum dispersal potential (Epopa et al. 2017; Verdonschot and Besse‐Lototskaya 2014; Yao et al. 2022).

We found statistically significant but weak support for isolation‐by‐distance (IBD) among samples from São Tomé Island, while no significant trend was observed for samples on Príncipe Island (Figure 5). Results from the spatial autocorrelation analyses suggest a dispersal range for *A. coluzzii* of approximately 7 km (Figure 6). This finding could explain the weak support for IBD, particularly on Príncipe Island, where the estimated dispersal range covers more than 70% of the pairwise distances between collection sites. In contrast, the complementary method used to measure the dispersal of *A. coluzzii* on these islands using *disperseNN2* CNN yielded lower estimates of 2.58 km for São Tomé and 2.83 km for Príncipe. Although these estimates differ, the CNN‐derived dispersal distances fall within the range of the highest values obtained from the spatial autocorrelation analysis. This discrepancy may highlight the influence of methodologies or assumptions inherent in each approach. While the spatial autocorrelation method suggests a broader potential dispersal range, the more conservative estimates provided by *disperseNN2* may reflect the geographic distribution and ecologic realities faced by *A. coluzzii* populations on the islands. This complexity underscores the importance of using multiple methods to gain a comprehensive understanding of dispersal dynamics in these vector populations.

We employed the EEMS method to identify specific regions where gene flow could potentially be reduced or facilitated. Notably, both islands have large national parks, encompassing approximately 23% and 42% of the total land area of São Tomé and Príncipe, respectively (Figure 1). These parks are unpopulated, and the terrain is rocky and steep factors disfavoring this highly anthropophilic and temporary water‐breeding mosquito species. We have explored both parks for the presence of *A. coluzzii* but found none. Consequently, we assumed that these parks would serve as barriers to dispersal and gene flow; however, this assumption was not strongly supported by the EEMS analysis (Figure 7). The EEMS analysis does indicate that, as expected, regions with higher human populations exhibit increased gene flow among populations within northeast São Tomé and central Príncipe (Figure 7D). The small size of Príncipe Island (Figure 1C), with an environment posing little resistance to gene flow (Figure 7), and a mosquito species with a dispersal capacity covering nearly the entire island (Figure 6A) potentially contribute to the observed lack of genetic structure, such that *A. coluzzii* on this island appears to represent a single Mendelian population.

Our interest is in the application of the data generated by this study to the design of a field trial release for a genetically engineered mosquito (GEM), in this case *A. coluzzii*. This GEM includes two anti‐parasite genes linked to a low‐threshold gene drive (Carballar‐Lejarazú et al. 2023). The goal is the elimination of *Plasmodium falciparum* from the islands by the introduction and spread of the gene construct such that its frequency reaches near 100% of the *A. coluzzii* on each island, therefore eliminating the parasite. There are two key issues that influence the efficacy of this approach. First, the number and location of the release sites, and second, the size of the natural populations of *A. coluzzii* on each island. In this study, we are focused on the first issue. It is desirable to conduct the fewest number of releases necessary because, in a larger scale deployment, the number of releases impacts cost and sustainability. It is anticipated that following initial introduction of the transgene at a release site it will be introduced across space, for example, from the release site into neighboring sites, via active GEM dispersal, and upon introduction will rapidly increase in frequency facilitated by the gene drive. It is advantageous to locate release sites in populations that have a high level of connectivity to neighboring sites to facilitate spread. The results presented here suggest that *A. coluzzii* on São Tomé and Príncipe Islands are significantly diverged from each other (Figure 2), but populations within each island are nearly panmictic (Figure 4 and Figure S4). However, some level of genetic structure is evident, and even weak barriers to gene flow (Figure 7) should be considered in selecting the number and location of release sites in a field trial design.

## Conflicts of Interest

The authors declare no conflicts of interest.

## Supporting information


Data S1.

## Data Availability

The sequence data that support the findings of this study have been deposited in GenBank with accession numbers SAMN25173698–SAMN25174013 and SAMN38765460–SAMN38765466 under BioProject ID PRJNA779397. Sample ID and corresponding accession numbers can be found in Table S1.
