# Supplementary material for: Patterns of Gene Flow in Anopheles coluzzii Populations From Two African Oceanic Islands
Source: Evol Appl. 2024 Nov 26;17(11):e70044. doi: 10.1111/eva.70044 (PMC11589655; doi:10.1111/eva.70044)

Supplementary Table 1 - Metadata for the Anopheles coluzzii specimens included in the study.

SampleID	Island	Code	Site Name	District	Year	Latitude	Longitude	Raw reads	Mapped reads	% Mapped	Coverage Mean	Accession Number
1204-ST-DIV-004	São Tomé	DIV	Diogo Vaz	MeZochi	2019	0.314567	6.651017	32547621	28052258	86.19%	12.13	SAMN25173906
1204-ST-DIV-005	São Tomé	DIV	Diogo Vaz	MeZochi	2019	0.314567	6.651017	25295784	23567364	93.17%	10.44	SAMN25173907
1204-ST-DIV-007	São Tomé	DIV	Diogo Vaz	MeZochi	2019	0.314567	6.651017	25736293	22252925	86.47%	9.56	SAMN25173908
1204-ST-DIV-014	São Tomé	DIV	Diogo Vaz	MeZochi	2019	0.314567	6.651017	37030241	35271989	95.25%	14.33	SAMN25173909
1204-ST-FED3-005	São Tomé	FED3	Fernao Dias	Lobata	2019	0.407867	6.66915	25594732	25140409	98.22%	11.08	SAMN25173910
1204-ST-FED3-007	São Tomé	FED3	Fernao Dias	Lobata	2019	0.407867	6.66915	29043361	28602579	98.48%	12.78	SAMN25173911
1204-ST-FED3-011	São Tomé	FED3	Fernao Dias	Lobata	2019	0.407867	6.66915	31736512	31136927	98.11%	13.74	SAMN25173912
1204-ST-FED3-014	São Tomé	FED3	Fernao Dias	Lobata	2019	0.407867	6.66915	34960348	34207160	97.85%	15.21	SAMN25173913
1204-ST-GDL-014	São Tomé	GDL	Guadalupe	Lobata	2019	0.3779	6.636933	25611838	24627032	96.15%	10.85	SAMN25173915
1204-ST-GDL-016	São Tomé	GDL	Guadalupe	Lobata	2019	0.3779	6.636933	28585349	25882471	90.54%	11.11	SAMN25173914
1204-ST-MIC-004	São Tomé	MIC	Micolo	Lobata	2019	0.40415	6.686983	26114482	25632533	98.15%	11.56	SAMN25173927
1204-ST-MIC-005	São Tomé	MIC	Micolo	Lobata	2019	0.40415	6.686983	29288093	28759410	98.19%	12.54	SAMN25173928
1204-ST-MIC-009	São Tomé	MIC	Micolo	Lobata	2019	0.40415	6.686983	29195999	28590799	97.93%	12.98	SAMN25173929
1204-ST-MIC-010	São Tomé	MIC	Micolo	Lobata	2019	0.40415	6.686983	35731286	35180416	98.46%	15.26	SAMN25173930
1204-ST-MRP-001	São Tomé	MRP	Morro Peixe	Lobata	2019	0.406383	6.642567	26407581	26029631	98.57%	11.93	SAMN25173939
1204-ST-MRP-006	São Tomé	MRP	Morro Peixe	Lobata	2019	0.406383	6.642567	36445710	35532235	97.49%	15.39	SAMN25173940
1204-ST-MRP-007	São Tomé	MRP	Morro Peixe	Lobata	2019	0.406383	6.642567	28570270	28300251	99.05%	12.48	SAMN25173941
1204-ST-MRP-011	São Tomé	MRP	Morro Peixe	Lobata	2019	0.406383	6.642567	30370791	29106017	95.84%	13.15	SAMN25173942
1204-ST-NEV-003	São Tomé	NEV	Neves	Lemba	2019	0.355033	6.546583	35832297	35481124	99.02%	15.36	SAMN25173951
1204-ST-NEV-006	São Tomé	NEV	Neves	Lemba	2019	0.355033	6.546583	32699989	32265189	98.67%	14.01	SAMN25173952
1204-ST-NEV-007	São Tomé	NEV	Neves	Lemba	2019	0.355033	6.546583	27096860	26573880	98.07%	11.71	SAMN25173953
1204-ST-NEV-011	São Tomé	NEV	Neves	Lemba	2019	0.355033	6.546583	34619136	33970989	98.13%	14.25	SAMN25173954
1204-ST-PFI3-001	São Tomé	PFI3	Ponta Figo	Lemba	2019	0.345667	6.544833	31053191	29211707	94.07%	13.33	SAMN25173959
1204-ST-PFI3-005	São Tomé	PFI3	Ponta Figo	Lemba	2019	0.345667	6.544833	29802488	28033342	94.06%	12.19	SAMN25173960
1204-ST-RLE-004	São Tomé	RLE	Roca Lemba	Lemba	2019	0.249883	6.4666	27782078	27477094	98.90%	12.36	SAMN25173989
1204-ST-RLE-010	São Tomé	RLE	Roca Lemba	Lemba	2019	0.249883	6.4666	60201957	58638510	97.40%	26.58	SAMN25173987
1204-ST-RLE-012	São Tomé	RLE	Roca Lemba	Lemba	2019	0.249883	6.4666	91419489	89976863	98.42%	40.00	SAMN25173988
1204-ST-RLE-014	São Tomé	RLE	Roca Lemba	Lemba	2019	0.249883	6.4666	29559722	29191888	98.76%	12.88	SAMN25173990
1204-ST-STC2-002	São Tomé	STC2	Santa Catarina	Lemba	2019	0.266767	6.46955	54157203	52767884	97.43%	23.58	SAMN25173999
1204-ST-STC2-003	São Tomé	STC2	Santa Catarina	Lemba	2019	0.266767	6.46955	55142553	54140660	98.18%	24.33	SAMN25174000
1204-ST-STC2-010	São Tomé	STC2	Santa Catarina	Lemba	2019	0.266767	6.46955	34756896	33745283	97.09%	15.15	SAMN25174001
1204-ST-STC2-012	São Tomé	STC2	Santa Catarina	Lemba	2019	0.266767	6.46955	37677319	37013669	98.24%	16.38	SAMN25174002
1205-ST-BOE-008	São Tomé	BOE	Boa Entrada	Lobata	2019	0.351717	6.6636	23557874	23316593	98.98%	10.61	SAMN25173882
1205-ST-BOE-011	São Tomé	BOE	Boa Entrada	Lobata	2019	0.351717	6.6636	34777469	34375716	98.84%	15.40	SAMN25173883
1205-ST-BOE-012	São Tomé	BOE	Boa Entrada	Lobata	2019	0.351717	6.6636	32861097	32426716	98.68%	14.61	SAMN25173884
1205-ST-BOE-013	São Tomé	BOE	Boa Entrada	Lobata	2019	0.351717	6.6636	35272467	34730974	98.46%	15.69	SAMN25173885
1205-ST-CON-003	São Tomé	CON	Conde	Lobata	2019	0.377266	6.66559	28407641	28004918	98.58%	12.67	SAMN25173898
1205-ST-CON-008	São Tomé	CON	Conde	Lobata	2019	0.377266	6.66559	35528700	35106512	98.81%	15.74	SAMN25173899
1205-ST-CON-012	São Tomé	CON	Conde	Lobata	2019	0.377266	6.66559	30049598	28024506	93.26%	11.49	SAMN25173900
1205-ST-CON-014	São Tomé	CON	Conde	Lobata	2019	0.377266	6.66559	29982909	28899649	96.39%	13.10	SAMN25173901
1206-ST-AZE-010	São Tomé	AZE	Agua Ize	Cantagalo	2019	0.220583	6.73075	23470572	23069256	98.29%	11.05	SAMN25173869
1206-ST-AZE-012	São Tomé	AZE	Agua Ize	Cantagalo	2019	0.220583	6.73075	35867499	35545019	99.10%	16.75	SAMN25173870
1206-ST-AZE-017	São Tomé	AZE	Agua Ize	Cantagalo	2019	0.220583	6.73075	31324059	30938176	98.77%	13.93	SAMN25173871
1206-ST-AZE-018	São Tomé	AZE	Agua Ize	Cantagalo	2019	0.220583	6.73075	33326339	33035453	99.13%	15.54	SAMN25173872
1206-ST-MEA-010	São Tomé	MEA	Mestro Antonio	Cantagalo	2019	0.25015	6.73175	70796347	69508645	98.18%	31.62	SAMN25173923
1206-ST-MEA-014	São Tomé	MEA	Mestro Antonio	Cantagalo	2019	0.25015	6.73175	32113517	31611313	98.44%	14.21	SAMN25173924
1206-ST-MEA-015	São Tomé	MEA	Mestro Antonio	Cantagalo	2019	0.25015	6.73175	31313232	30680445	97.98%	13.83	SAMN25173925
1206-ST-MEA-017	São Tomé	MEA	Mestro Antonio	Cantagalo	2019	0.25015	6.73175	30565992	30024691	98.23%	13.54	SAMN25173926
1206-ST-NDA-001	São Tomé	NDA	Nova Olinda	Cantagalo	2019	0.240767	6.733567	25734825	25322533	98.40%	11.65	SAMN25173947
1206-ST-NDA-003	São Tomé	NDA	Nova Olinda	Cantagalo	2019	0.240767	6.733567	22711909	22389843	98.58%	10.29	SAMN25173948
1206-ST-NDA-005	São Tomé	NDA	Nova Olinda	Cantagalo	2019	0.240767	6.733567	25808402	25372616	98.31%	11.71	SAMN25173949
1206-ST-NDA-007	São Tomé	NDA	Nova Olinda	Cantagalo	2019	0.240767	6.733567	30665338	30152176	98.33%	13.65	SAMN25173950
1206-ST-RBA-001	São Tomé	RBA	Ribeira Alfonso	Cantagalo	2019	0.194483	6.69915	27600879	27342026	99.06%	12.07	SAMN25173983
1206-ST-RBA-003	São Tomé	RBA	Ribeira Alfonso	Cantagalo	2019	0.194483	6.69915	33477268	32964801	98.47%	14.76	SAMN25173984
1206-ST-RBA-004	São Tomé	RBA	Ribeira Alfonso	Cantagalo	2019	0.194483	6.69915	56578292	55936338	98.87%	25.38	SAMN25173985
1206-ST-RBA-009	São Tomé	RBA	Ribeira Alfonso	Cantagalo	2019	0.194483	6.69915	31368251	30330220	96.69%	13.64	SAMN25173986
1206-ST-SNA-001	São Tomé	SNA	Santa Ana	Cantagalo	2019	0.256533	6.74255	51205586	50490720	98.60%	22.71	SAMN25173995
1206-ST-SNA-004	São Tomé	SNA	Santa Ana	Cantagalo	2019	0.256533	6.74255	30590846	30348220	99.21%	13.87	SAMN25173996
1206-ST-SNA-005	São Tomé	SNA	Santa Ana	Cantagalo	2019	0.256533	6.74255	28506123	27084726	95.01%	11.90	SAMN25173997
1206-ST-SNA-006	São Tomé	SNA	Santa Ana	Cantagalo	2019	0.256533	6.74255	28493458	27125310	95.20%	12.02	SAMN25173998
1206-ST-UBU-002	São Tomé	UBU	Uba Budu	Cantagalo	2019	0.2693	6.746633	30100649	28875877	95.93%	13.17	SAMN25174010
1206-ST-UBU-003	São Tomé	UBU	Uba Budu	Cantagalo	2019	0.2693	6.746633	28360972	27893589	98.35%	12.70	SAMN25174011
1206-ST-UBU-007	São Tomé	UBU	Uba Budu	Cantagalo	2019	0.2693	6.746633	28715767	27785728	96.76%	12.47	SAMN25174012
1206-ST-UBU-014	São Tomé	UBU	Uba Budu	Cantagalo	2019	0.2693	6.746633	32737701	31889212	97.41%	14.43	SAMN25174013
1207-ST-CDF-001	São Tomé	CDF	Claudino Faro	Cantagalo	2019	0.23845	6.652217	36005759	35650938	99.01%	15.69	SAMN25173886
1207-ST-CDF-003	São Tomé	CDF	Claudino Faro	Cantagalo	2019	0.23845	6.652217	34791573	34386701	98.84%	15.23	SAMN25173887
1207-ST-CDF-005	São Tomé	CDF	Claudino Faro	Cantagalo	2019	0.23845	6.652217	30216267	29848135	98.78%	13.46	SAMN25173888
1207-ST-CDF-008	São Tomé	CDF	Claudino Faro	Cantagalo	2019	0.23845	6.652217	31241374	30881057	98.85%	13.50	SAMN25173889
1207-ST-MTC-002	São Tomé	MTC	Mato Cana	Cantagalo	2019	0.2322	6.702483	37490560	37013822	98.73%	16.66	SAMN25173943
1207-ST-MTC-003	São Tomé	MTC	Mato Cana	Cantagalo	2019	0.2322	6.702483	34591556	34200668	98.87%	15.48	SAMN25173944
1207-ST-MTC-004	São Tomé	MTC	Mato Cana	Cantagalo	2019	0.2322	6.702483	37222275	36794767	98.85%	16.59	SAMN25173945
1207-ST-MTC-005	São Tomé	MTC	Mato Cana	Cantagalo	2019	0.2322	6.702483	37959038	37578761	99.00%	16.87	SAMN25173946
1209-ST-CLO-001	São Tomé	CLO	Clotilde	Caue	2019	0.110467	6.598233	35117724	34826238	99.17%	15.58	SAMN25173894
1209-ST-CLO-002	São Tomé	CLO	Clotilde	Caue	2019	0.110467	6.598233	29891481	29622014	99.10%	13.16	SAMN25173895
1209-ST-CLO-005	São Tomé	CLO	Clotilde	Caue	2019	0.110467	6.598233	25257173	25009920	99.02%	11.20	SAMN25173896
1209-ST-CLO-012	São Tomé	CLO	Clotilde	Caue	2019	0.110467	6.598233	33713083	33389111	99.04%	14.67	SAMN25173897
1209-ST-MAL-003	São Tomé	MAL	Malanza	Caue	2019	0.048783	6.535267	28725922	28455886	99.06%	12.85	SAMN25173919
1209-ST-MAL-007	São Tomé	MAL	Malanza	Caue	2019	0.048783	6.535267	37007912	36729756	99.25%	16.01	SAMN25173920
1209-ST-MAL-013	São Tomé	MAL	Malanza	Caue	2019	0.048783	6.535267	30140231	29777169	98.80%	13.53	SAMN25173921

1209-ST-MAL-014	São Tomé	MAL	Malanza	Caue	2019	0.048783	6.535267	27624420	26707352	96.68%	11.61	SAMN25173922
1209-ST-MOM-002	São Tomé	MOM	Monte Mario	Caue	2019	0.070217	6.563117	34993834	34608381	98.90%	14.80	SAMN25173935
1209-ST-MOM-004	São Tomé	MOM	Monte Mario	Caue	2019	0.070217	6.563117	39098553	38123133	97.51%	16.66	SAMN25173936
1209-ST-MOM-005	São Tomé	MOM	Monte Mario	Caue	2019	0.070217	6.563117	35927783	35076443	97.63%	15.18	SAMN25173937
1209-ST-MOM-007	São Tomé	MOM	Monte Mario	Caue	2019	0.070217	6.563117	26573630	25609999	96.37%	11.63	SAMN25173938
1209-ST-POA1-001	São Tomé	POA1	Porto Alegre	Caue	2019	0.034967	6.534783	29816022	29586941	99.23%	13.16	SAMN25173977
1209-ST-POA1-004	São Tomé	POA1	Porto Alegre	Caue	2019	0.034967	6.534783	34063098	33694406	98.92%	15.20	SAMN25173978
1209-ST-POA1-005	São Tomé	POA1	Porto Alegre	Caue	2019	0.034967	6.534783	31453045	31157245	99.06%	14.39	SAMN25173979
1209-ST-POA1-007	São Tomé	POA1	Porto Alegre	Caue	2019	0.034967	6.534783	31541086	31168449	98.82%	14.28	SAMN25173980
1210-ST-LUC-001	São Tomé	LUC	Lucumi	AguaGrande	2019	0.33455	6.7283	35495617	35089587	98.86%	15.65	SAMN25173916
1210-ST-LUC-003	São Tomé	LUC	Lucumi	AguaGrande	2019	0.33455	6.7283	36630083	35958732	98.17%	15.75	SAMN25173917
1210-ST-LUC-004	São Tomé	LUC	Lucumi	AguaGrande	2019	0.33455	6.7283	30712425	30399203	98.98%	13.38	SAMN25173918
1210-ST-MOC-001	São Tomé	MOC	Monte Cafe	MeZochi	2019	0.300833	6.639183	25831697	24648885	95.42%	11.12	SAMN25173933
1210-ST-MOC-002	São Tomé	MOC	Monte Cafe	MeZochi	2019	0.300833	6.639183	27598217	26329447	95.40%	11.95	SAMN25173931
1210-ST-MOC-003	São Tomé	MOC	Monte Cafe	MeZochi	2019	0.300833	6.639183	25543432	24684093	96.64%	11.21	SAMN25173932
1210-ST-MOC-010	São Tomé	MOC	Monte Cafe	MeZochi	2019	0.300833	6.639183	34783128	32854512	94.46%	14.72	SAMN25173934
1210-ST-TRD-001	São Tomé	TRD	Torres Dias	MeZochi	2019	0.3147	6.700383	30956974	30667484	99.06%	13.70	SAMN25174003
1210-ST-TRD-005	São Tomé	TRD	Torres Dias	MeZochi	2019	0.3147	6.700383	27325568	27074411	99.08%	12.14	SAMN25174004
1210-ST-TRD-006	São Tomé	TRD	Torres Dias	MeZochi	2019	0.3147	6.700383	26285685	26059865	99.14%	11.81	SAMN25174005
1210-ST-TRD-008	São Tomé	TRD	Torres Dias	MeZochi	2019	0.3147	6.700383	32421492	31245946	96.37%	14.14	SAMN25174006
1211-ST-ANG-004	São Tomé	ANG	Angolares	Caue	2019	0.134117	6.648233	24843121	19511840	78.54%	8.77	SAMN25173860
1211-ST-ANG-005	São Tomé	ANG	Angolares	Caue	2019	0.134117	6.648233	36738995	30239356	82.31%	14.00	SAMN25173861
1211-ST-ANG-006	São Tomé	ANG	Angolares	Caue	2019	0.134117	6.648233	23221248	20523998	88.38%	9.23	SAMN25173862
1211-ST-ANG-007	São Tomé	ANG	Angolares	Caue	2019	0.134117	6.648233	38039204	24925529	65.53%	11.68	SAMN25173863
1211-ST-ANG-008	São Tomé	ANG	Angolares	Caue	2019	0.134117	6.648233	45036428	42607468	94.61%	19.34	SAMN25173864
1211-ST-ATP-001	São Tomé	ATP	Angra Toldo	Caue	2019	0.1577	6.670583	35298159	34963161	99.05%	16.44	SAMN25173865
1211-ST-ATP-005	São Tomé	ATP	Angra Toldo	Caue	2019	0.1577	6.670583	36017773	35396211	98.27%	16.02	SAMN25173866
1211-ST-ATP-006	São Tomé	ATP	Angra Toldo	Caue	2019	0.1577	6.670583	31696327	30855332	97.35%	13.91	SAMN25173867
1211-ST-ATP-008	São Tomé	ATP	Angra Toldo	Caue	2019	0.1577	6.670583	33632428	31928283	94.93%	14.35	SAMN25173868
1219-ST-ALM-001	São Tomé	ALM	Almas	MeZochi	2019	0.300783	6.739633	31602860	31205724	98.74%	14.66	SAMN25173856
1219-ST-ALM-002	São Tomé	ALM	Almas	MeZochi	2019	0.300783	6.739633	32860238	32427684	98.68%	15.08	SAMN25173857
1219-ST-ALM-003	São Tomé	ALM	Almas	MeZochi	2019	0.300783	6.739633	34887635	34485868	98.85%	16.14	SAMN25173858
1219-ST-ALM-005	São Tomé	ALM	Almas	MeZochi	2019	0.300783	6.739633	38724507	38295943	98.89%	17.94	SAMN25173859
1219-ST-CDM2-005	São Tomé	CDM2	Compo de Milho	AguaGrande	2019	0.354417	6.7146	27019739	26187320	96.92%	10.67	SAMN25173890
1219-ST-CDM2-015	São Tomé	CDM2	Compo de Milho	AguaGrande	2019	0.354417	6.7146	29862037	29229206	97.88%	11.69	SAMN25173893
1219-ST-CDM2-016	São Tomé	CDM2	Compo de Milho	AguaGrande	2019	0.354417	6.7146	26262756	25898172	98.61%	10.54	SAMN25173891
1219-ST-CDM2-017	São Tomé	CDM2	Compo de Milho	AguaGrande	2019	0.354417	6.7146	30990620	30633741	98.85%	12.26	SAMN25173892
1219-ST-CRU-043	São Tomé	CRU	Cruzeiro	MeZochi	2019	0.2905	6.6813	28685028	28420313	99.08%	12.80	SAMN25173902
1219-ST-CRU-044	São Tomé	CRU	Cruzeiro	MeZochi	2019	0.2905	6.6813	26036714	25491176	97.90%	10.97	SAMN25173903
1219-ST-CRU-046	São Tomé	CRU	Cruzeiro	MeZochi	2019	0.2905	6.6813	28837936	28406718	98.50%	12.11	SAMN25173905
1219-ST-CRU-047	São Tomé	CRU	Cruzeiro	MeZochi	2019	0.2905	6.6813	31389876	30440916	96.98%	13.69	SAMN25173904
1219-ST-PAN-001	São Tomé	PAN	Pantufo	MeZochi	2019	0.31495	6.743233	31355210	30024630	95.76%	13.71	SAMN25173955
1219-ST-PAN-003	São Tomé	PAN	Pantufo	MeZochi	2019	0.31495	6.743233	29995809	29432934	98.12%	13.69	SAMN25173956
1219-ST-PAN-008	São Tomé	PAN	Pantufo	MeZochi	2019	0.31495	6.743233	28380518	27706683	97.63%	12.53	SAMN25173957
1219-ST-PAN-009	São Tomé	PAN	Pantufo	MeZochi	2019	0.31495	6.743233	39320197	38616448	98.21%	16.82	SAMN25173958
1219-ST-PGA-012	São Tomé	PGA	Praia Gamboa	AguaGrande	2019	0.3798	6.71485	30717222	30044890	97.81%	13.79	SAMN25173961
1219-ST-PGA-014	São Tomé	PGA	Praia Gamboa	AguaGrande	2019	0.3798	6.71485	30729925	29604096	96.34%	13.57	SAMN25173962
1219-ST-PGA-016	São Tomé	PGA	Praia Gamboa	AguaGrande	2019	0.3798	6.71485	25377073	25068277	98.78%	11.48	SAMN25173963
1219-ST-PGA-017	São Tomé	PGA	Praia Gamboa	AguaGrande	2019	0.3798	6.71485	28259417	27696218	98.01%	12.52	SAMN25173964
1219-ST-PIN-007	São Tomé	PIN	Pinheira	Cantagalo	2019	0.296017	6.743683	32801542	32294626	98.45%	14.90	SAMN25173973
1219-ST-PIN-008	São Tomé	PIN	Pinheira	Cantagalo	2019	0.296017	6.743683	30490620	29971831	98.30%	13.79	SAMN25173974
1219-ST-PIN-009	São Tomé	PIN	Pinheira	Cantagalo	2019	0.296017	6.743683	30974188	30434996	98.26%	13.97	SAMN25173975
1219-ST-PIN-010	São Tomé	PIN	Pinheira	Cantagalo	2019	0.296017	6.743683	26589788	26197345	98.52%	12.12	SAMN25173976
1219-ST-PRM-001	São Tomé	PRM	Praia Melao	MeZochi	2019	0.305367	6.74875	27764954	27345117	98.49%	12.62	SAMN25173981
1219-ST-PRM-005	São Tomé	PRM	Praia Melao	MeZochi	2019	0.305367	6.74875	72213201	70639155	97.82%	31.91	SAMN25173982
1219-ST-SMA-009	São Tomé	SMA	Sao Marcal	AguaGrande	2019	0.321583	6.736183	35748830	34749427	97.20%	14.31	SAMN25173991
1219-ST-SMA-010	São Tomé	SMA	Sao Marcal	AguaGrande	2019	0.321583	6.736183	32069825	31308728	97.63%	13.28	SAMN25173992
1219-ST-SMA-011	São Tomé	SMA	Sao Marcal	AguaGrande	2019	0.321583	6.736183	33632996	33131257	98.51%	13.84	SAMN25173993
1219-ST-SMA-024	São Tomé	SMA	Sao Marcal	AguaGrande	2019	0.321583	6.736183	42889237	42064375	98.08%	17.39	SAMN25173994
1219-ST-TRI-009	São Tomé	TRI	Trinidade	MeZochi	2019	0.296167	6.6798	67682796	66454239	98.18%	29.91	SAMN25174007
1219-ST-TRI-012	São Tomé	TRI	Trinidade	MeZochi	2019	0.296167	6.6798	39168513	38762616	98.96%	17.82	SAMN25174008
1219-ST-TRI-014	São Tomé	TRI	Trinidade	MeZochi	2019	0.296167	6.6798	36472643	36055098	98.86%	15.76	SAMN25174009
0322-PR-PCAR-2.1	Príncipe	PCAR	Praia Cara	Pague	2022	1.55774	7.34602	31844221	31548417	99.07%	14.00	SAMN38765460
0322-PR-PSEC-14.2	Príncipe	PSEC	Praia Seca	Pague	2022	1.54601	7.39981	14728448	14543064	98.74%	6.65	SAMN38765461
0322-PR-PSEC-15.2	Príncipe	PSEC	Praia Seca	Pague	2022	1.54601	7.39981	29240770	28825559	98.58%	13.10	SAMN38765462
0322-PR-PSEC-16.2	Príncipe	PSEC	Praia Seca	Pague	2022	1.54601	7.39981	23139326	22904244	98.98%	10.42	SAMN38765463
0322-PR-PSEC-17.1	Príncipe	PSEC	Praia Seca	Pague	2022	1.54601	7.39981	33063426	32481962	98.24%	14.61	SAMN38765464
0322-PR-PSEC-18.5	Príncipe	PSEC	Praia Seca	Pague	2022	1.54601	7.39981	31674525	31193908	98.48%	14.19	SAMN38765465
0322-PR-PSEC-19.2	Príncipe	PSEC	Praia Seca	Pague	2022	1.54601	7.39981	28592628	27894483	97.56%	12.68	SAMN38765466
1219-PR-AIR-001	Príncipe	AIR	Aeroporto	Pague	2019	1.6667	7.412267	33004711	32755148	99.24%	14.65	SAMN25173698
1219-PR-AIR-008	Príncipe	AIR	Aeroporto	Pague	2019	1.6667	7.412267	30498222	30263652	99.23%	13.82	SAMN25173699
1219-PR-AIR-013	Príncipe	AIR	Aeroporto	Pague	2019	1.6667	7.412267	28954135	28613339	98.82%	13.11	SAMN25173700
1219-PR-AIR-014	Príncipe	AIR	Aeroporto	Pague	2019	1.6667	7.412267	24820285	24446321	98.49%	11.33	SAMN25173701
1219-PR-AJT-002	Príncipe	AJT	Alojamento	Pague	2019	1.638267	7.416683	29658508	29304879	98.81%	13.42	SAMN25173702
1219-PR-AJT-003	Príncipe	AJT	Alojamento	Pague	2019	1.638267	7.416683	26772092	26491305	98.95%	11.78	SAMN25173703
1219-PR-AJT-006	Príncipe	AJT	Alojamento	Pague	2019	1.638267	7.416683	30861396	29670723	96.14%	13.14	SAMN25173704
1219-PR-AJT-013	Príncipe	AJT	Alojamento	Pague	2019	1.638267	7.416683	30042619	29035326	96.65%	12.98	SAMN25173705
1219-PR-AZT-004	Príncipe	AZT	Azeitona	Pague	2019	1.664633	7.4025	27132989	26788538	98.73%	12.27	SAMN25173706
1219-PR-AZT-005	Príncipe	AZT	Azeitona	Pague	2019	1.664633	7.4025	31021203	30650641	98.81%	13.78	SAMN25173707
1219-PR-AZT-010	Príncipe	AZT	Azeitona	Pague	2019	1.664633	7.4025	31612258	31160050	98.57%	14.02	SAMN25173708
1219-PR-AZT-016	Príncipe	AZT	Azeitona	Pague	2019	1.664633	7.4025	30290420	29718200	98.11%	11.82	SAMN25173709
1219-PR-BLV-007	Príncipe	BLV	Bela Vista	Pague	2019	1.617567	7.413583	25809171	25608776	99.22%	11.45	SAMN25173710

1219-PR-BLV-008	Príncipe	BLV	Bela Vista	Pague	2019	1.617567	7.413583	30429316	30208234	99.27%	13.58	SAMN25173711
1219-PR-BLV-018	Príncipe	BLV	Bela Vista	Pague	2019	1.617567	7.413583	31630788	30678013	96.99%	13.69	SAMN25173712
1219-PR-BLV-019	Príncipe	BLV	Bela Vista	Pague	2019	1.617567	7.413583	28796786	27537255	95.63%	12.58	SAMN25173713
1219-PR-BMT-007	Príncipe	BMT	Belo Monte	Pague	2019	1.681867	7.443917	25152734	24751103	98.40%	11.19	SAMN25173714
1219-PR-BMT-010	Príncipe	BMT	Belo Monte	Pague	2019	1.681867	7.443917	28480321	27990728	98.28%	12.46	SAMN25173715
1219-PR-BMT-012	Príncipe	BMT	Belo Monte	Pague	2019	1.681867	7.443917	22654835	22090789	97.51%	10.05	SAMN25173716
1219-PR-BOM-001	Príncipe	BOM	Bombom	Pague	2019	1.688983	7.402067	44007457	43535540	98.93%	19.52	SAMN25173717
1219-PR-BOM-003	Príncipe	BOM	Bombom	Pague	2019	1.688983	7.402067	48720227	48139701	98.81%	21.56	SAMN25173718
1219-PR-BOM-018	Príncipe	BOM	Bombom	Pague	2019	1.688983	7.402067	38873488	38153562	98.15%	17.26	SAMN25173719
1219-PR-BOM-019	Príncipe	BOM	Bombom	Pague	2019	1.688983	7.402067	33380345	32929562	98.65%	14.65	SAMN25173720
1219-PR-GAS-006	Príncipe	GAS	Gaspar	Pague	2019	1.647967	7.41175	30090662	29731217	98.81%	13.07	SAMN25173721
1219-PR-GAS-007	Príncipe	GAS	Gaspar	Pague	2019	1.647967	7.41175	24275142	24029680	98.99%	10.84	SAMN25173722
1219-PR-GAS-010	Príncipe	GAS	Gaspar	Pague	2019	1.647967	7.41175	39312543	38671528	98.37%	17.48	SAMN25173723
1219-PR-GAS-011	Príncipe	GAS	Gaspar	Pague	2019	1.647967	7.41175	28033449	27478940	98.02%	12.37	SAMN25173724
1219-PR-NVE2-001	Príncipe	NVE2	Nova Estrela	Pague	2019	1.616333	7.428667	27688077	27422257	99.04%	12.46	SAMN25173725
1219-PR-NVE2-002	Príncipe	NVE2	Nova Estrela	Pague	2019	1.616333	7.428667	25997016	25755837	99.07%	11.80	SAMN25173726
1219-PR-NVE2-003	Príncipe	NVE2	Nova Estrela	Pague	2019	1.616333	7.428667	27567849	27302001	99.04%	12.55	SAMN25173727
1219-PR-NVE2-008	Príncipe	NVE2	Nova Estrela	Pague	2019	1.616333	7.428667	31814897	31478773	98.94%	14.55	SAMN25173728
1219-PR-PAC-001	Príncipe	PAC	Paciencia	Pague	2019	1.669733	7.431333	35639908	35304333	99.06%	15.97	SAMN25173729
1219-PR-PAC-003	Príncipe	PAC	Paciencia	Pague	2019	1.669733	7.431333	30445665	30159860	99.06%	13.81	SAMN25173730
1219-PR-PAC-004	Príncipe	PAC	Paciencia	Pague	2019	1.669733	7.431333	27806723	27528618	99.00%	12.66	SAMN25173731
1219-PR-PAC-007	Príncipe	PAC	Paciencia	Pague	2019	1.669733	7.431333	27549001	26423552	95.91%	11.88	SAMN25173732
1219-PR-PAD-001	Príncipe	PAD	Praia Abade	Pague	2019	1.63015	7.4575	27437372	26939272	98.18%	11.98	SAMN25173733
1219-PR-PAD-006	Príncipe	PAD	Praia Abade	Pague	2019	1.63015	7.4575	22687736	22221503	97.94%	9.88	SAMN25173734
1219-PR-PAD-008	Príncipe	PAD	Praia Abade	Pague	2019	1.63015	7.4575	25857830	25194309	97.43%	11.28	SAMN25173735
1219-PR-PAD-010	Príncipe	PAD	Praia Abade	Pague	2019	1.63015	7.4575	27835685	27317945	98.14%	12.35	SAMN25173736
1219-PR-PBA-005	Príncipe	PBA	Praia Banana	Pague	2019	1.69025	7.442183	26633771	26023744	97.71%	11.31	SAMN25173737
1219-PR-PBA-006	Príncipe	PBA	Praia Banana	Pague	2019	1.69025	7.442183	29484163	29205917	99.06%	13.15	SAMN25173738
1219-PR-PBA-007	Príncipe	PBA	Praia Banana	Pague	2019	1.69025	7.442183	28527154	28175178	98.77%	12.62	SAMN25173739
1219-PR-PBA-008	Príncipe	PBA	Praia Banana	Pague	2019	1.69025	7.442183	28969775	28704718	99.09%	12.88	SAMN25173740
1219-PR-PBT-001	Príncipe	PBT	Ponte Bareto	Pague	2019	1.63185	7.4172	23716237	23502379	99.10%	10.75	SAMN25173741
1219-PR-PBT-002	Príncipe	PBT	Ponte Bareto	Pague	2019	1.63185	7.4172	30309554	30063965	99.19%	13.45	SAMN25173742
1219-PR-PBT-003	Príncipe	PBT	Ponte Bareto	Pague	2019	1.63185	7.4172	30014261	29810709	99.32%	13.52	SAMN25173743
1219-PR-PBT-005	Príncipe	PBT	Ponte Bareto	Pague	2019	1.63185	7.4172	27873335	27558949	98.87%	12.65	SAMN25173744
1219-PR-PBU-003	Príncipe	PBU	Praia Burra	Pague	2019	1.682817	7.43495	29191362	28915918	99.06%	12.87	SAMN25173745
1219-PR-PBU-004	Príncipe	PBU	Praia Burra	Pague	2019	1.682817	7.43495	24657371	24308103	98.58%	10.61	SAMN25173746
1219-PR-PBU-007	Príncipe	PBU	Praia Burra	Pague	2019	1.682817	7.43495	26049503	25408200	97.54%	11.37	SAMN25173747
1219-PR-PBU-010	Príncipe	PBU	Praia Burra	Pague	2019	1.682817	7.43495	32736501	32202840	98.37%	14.67	SAMN25173748
1219-PR-PCA-002	Príncipe	PCA	Praia Campanha	Pague	2019	1.684067	7.425217	35859277	35418665	98.77%	14.07	SAMN25173749
1219-PR-PCA-004	Príncipe	PCA	Praia Campanha	Pague	2019	1.684067	7.425217	23324708	23070964	98.91%	10.45	SAMN25173750
1219-PR-PCA-007	Príncipe	PCA	Praia Campanha	Pague	2019	1.684067	7.425217	26093010	25774061	98.78%	11.57	SAMN25173751
1219-PR-PCA-016	Príncipe	PCA	Praia Campanha	Pague	2019	1.684067	7.425217	27610726	27230080	98.62%	11.16	SAMN25173752
1219-PR-PDS-002	Príncipe	PDS	Ponta do Sol	Pague	2019	1.655333	7.38125	28258963	28042214	99.23%	12.69	SAMN25173753
1219-PR-PDS-003	Príncipe	PDS	Ponta do Sol	Pague	2019	1.655333	7.38125	31295124	31042396	99.19%	14.12	SAMN25173754
1219-PR-PDS-006	Príncipe	PDS	Ponta do Sol	Pague	2019	1.655333	7.38125	27926020	26951146	96.51%	11.89	SAMN25173755
1219-PR-PDS-008	Príncipe	PDS	Ponta do Sol	Pague	2019	1.655333	7.38125	25020735	24752995	98.93%	10.91	SAMN25173756
1219-PR-PIA-006	Príncipe	PIA	Lenta Pia	Pague	2019	1.6397	7.418517	28333428	28050690	99.00%	11.40	SAMN25173757
1219-PR-PIA-009	Príncipe	PIA	Lenta Pia	Pague	2019	1.6397	7.418517	30269150	30006945	99.13%	14.27	SAMN25173758
1219-PR-PIA-014	Príncipe	PIA	Lenta Pia	Pague	2019	1.6397	7.418517	34188618	33811272	98.90%	15.76	SAMN25173759
1219-PR-PIA-017	Príncipe	PIA	Lenta Pia	Pague	2019	1.6397	7.418517	28941100	28247977	97.61%	12.50	SAMN25173760
1219-PR-PIC-004	Príncipe	PIC	Picao	Pague	2019	1.663417	7.42855	22988167	22450812	97.66%	10.15	SAMN25173761
1219-PR-PIC-005	Príncipe	PIC	Picao	Pague	2019	1.663417	7.42855	37408079	36253101	96.91%	17.06	SAMN25173762
1219-PR-PIC-011	Príncipe	PIC	Picao	Pague	2019	1.663417	7.42855	41855990	39697695	94.84%	16.98	SAMN25173763
1219-PR-PIH-007	Príncipe	PIH	Praia Ilhame	Pague	2019	1.661383	7.428517	26181976	25867488	98.80%	11.55	SAMN25173764
1219-PR-PIH-009	Príncipe	PIH	Praia Ilhame	Pague	2019	1.661383	7.428517	26864983	26620539	99.09%	11.85	SAMN25173765
1219-PR-PIH-018	Príncipe	PIH	Praia Ilhame	Pague	2019	1.661383	7.428517	30453957	30193032	99.14%	13.69	SAMN25173766
1219-PR-PIH-023	Príncipe	PIH	Praia Ilhame	Pague	2019	1.661383	7.428517	27760475	27482289	99.00%	12.08	SAMN25173767
1219-PR-PLAP-007	Príncipe	PLAP	Praia Lapa	Pague	2019	1.606133	7.364233	32128734	31825773	99.06%	13.94	SAMN25173768
1219-PR-PLAP-009	Príncipe	PLAP	Praia Lapa	Pague	2019	1.606133	7.364233	25176994	24942603	99.07%	11.19	SAMN25173769
1219-PR-PLAP-013	Príncipe	PLAP	Praia Lapa	Pague	2019	1.606133	7.364233	30499978	30243964	99.16%	13.29	SAMN25173770
1219-PR-PLAP-018	Príncipe	PLAP	Praia Lapa	Pague	2019	1.606133	7.364233	26442740	26176112	98.99%	11.73	SAMN25173771
1219-PR-POR-002	Príncipe	POR	Porto Real	Pague	2019	1.624617	7.406033	26850518	26580207	98.99%	11.62	SAMN25173772
1219-PR-POR-004	Príncipe	POR	Porto Real	Pague	2019	1.624617	7.406033	32461989	32163220	99.08%	14.40	SAMN25173773
1219-PR-POR-006	Príncipe	POR	Porto Real	Pague	2019	1.624617	7.406033	26687431	26421098	99.00%	11.88	SAMN25173774
1219-PR-POR-007	Príncipe	POR	Porto Real	Pague	2019	1.624617	7.406033	32613435	32203102	98.74%	14.03	SAMN25173775
1219-PR-PRMA-004	Príncipe	PRMA	Praia Macaco	Pague	2019	1.681567	7.453133	29899901	29520333	98.73%	13.57	SAMN25173785
1219-PR-PRMA-005	Príncipe	PRMA	Praia Macaco	Pague	2019	1.681567	7.453133	26532661	26259989	98.97%	11.54	SAMN25173786
1219-PR-PRMA-007	Príncipe	PRMA	Praia Macaco	Pague	2019	1.681567	7.453133	26068219	25754494	98.80%	11.13	SAMN25173787
1219-PR-PSE-002	Príncipe	PSE	Praia Seabra	Pague	2019	1.688867	7.4121	28782856	28397850	98.66%	12.70	SAMN25173788
1219-PR-PSE-003	Príncipe	PSE	Praia Seabra	Pague	2019	1.688867	7.4121	29728418	29330799	98.66%	13.15	SAMN25173789
1219-PR-PSE-007	Príncipe	PSE	Praia Seabra	Pague	2019	1.688867	7.4121	31205701	30879488	98.95%	13.91	SAMN25173790
1219-PR-PSE-008	Príncipe	PSE	Praia Seabra	Pague	2019	1.688867	7.4121	31424976	30984824	98.60%	13.88	SAMN25173791
1219-PR-RAD-001	Príncipe	RAD	Roca Abade	Pague	2019	1.625283	7.457267	28831378	28526879	98.94%	12.19	SAMN25173792
1219-PR-RAD-005	Príncipe	RAD	Roca Abade	Pague	2019	1.625283	7.457267	27407018	27157268	99.09%	11.77	SAMN25173793
1219-PR-RAD-006	Príncipe	RAD	Roca Abade	Pague	2019	1.625283	7.457267	28620455	28348347	99.05%	12.06	SAMN25173794
1219-PR-RAD-028	Príncipe	RAD	Roca Abade	Pague	2019	1.625283	7.457267	32936824	32053292	97.32%	14.69	SAMN25173795
1219-PR-RBF-003	Príncipe	RBF	Ribeira Fria	Pague	2019	1.590583	7.422133	26613638	26272436	98.72%	11.78	SAMN25173796
1219-PR-RBF2-014	Príncipe	RBF2	Ribeira Fria	Pague	2019	1.58825	7.423017	29470549	29142619	98.89%	13.01	SAMN25173797
1219-PR-RBF2-015	Príncipe	RBF2	Ribeira Fria	Pague	2019	1.58825	7.423017	28826099	28483109	98.81%	12.81	SAMN25173800
1219-PR-RBF2-016	Príncipe	RBF2	Ribeira Fria	Pague	2019	1.58825	7.423017	32085653	31798786	99.11%	14.08	SAMN25173798
1219-PR-RBF2-017	Príncipe	RBF2	Ribeira Fria	Pague	2019	1.58825	7.423017	34102861	33836759	99.22%	15.27	SAMN25173799
1219-PR-REZ-003	Príncipe	REZ	Ribeira Ize	Pague	2019	1.685483	7.3956	26115689	25911042	99.22%	11.82	SAMN25173801

1219-PR-REZ-005	Príncipe	REZ	Ribeira Ize	Pague	2019	1.685483	7.3956	30690914	30190138	98.37%	13.40	SAMN25173802
1219-PR-REZ-007	Príncipe	REZ	Ribeira Ize	Pague	2019	1.685483	7.3956	34721481	33739952	97.17%	14.72	SAMN25173803
1219-PR-REZ-014	Príncipe	REZ	Ribeira Ize	Pague	2019	1.685483	7.3956	26105613	25588159	98.02%	11.45	SAMN25173804
1219-PR-RFO-001	Príncipe	RFO	Ribeira Formiga	Pague	2019	1.640133	7.41735	31309085	31001159	99.02%	13.39	SAMN25173805
1219-PR-RFO-002	Príncipe	RFO	Ribeira Formiga	Pague	2019	1.640133	7.41735	32182330	31887352	99.08%	13.80	SAMN25173806
1219-PR-RFO-004	Príncipe	RFO	Ribeira Formiga	Pague	2019	1.640133	7.41735	31330080	31037553	99.07%	13.43	SAMN25173807
1219-PR-RFO-022	Príncipe	RFO	Ribeira Formiga	Pague	2019	1.640133	7.41735	34056388	33606369	98.68%	14.39	SAMN25173808
1219-PR-SAJ-005	Príncipe	SAJ	Sao Joao	Pague	2019	1.6428	7.43235	27736640	27364836	98.66%	12.49	SAMN25173820
1219-PR-SAJ-006	Príncipe	SAJ	Sao Joao	Pague	2019	1.6428	7.43235	32865859	32278386	98.21%	14.52	SAMN25173821
1219-PR-SAJ-007	Príncipe	SAJ	Sao Joao	Pague	2019	1.6428	7.43235	27202381	26957329	99.10%	12.24	SAMN25173822
1219-PR-SAJ-008	Príncipe	SAJ	Sao Joao	Pague	2019	1.6428	7.43235	27531603	27188898	98.76%	12.43	SAMN25173823
1219-PR-SAJL-007	Príncipe	SAJL	Sao Joao Largo	Pague	2019	1.64235	7.431867	31265855	30814416	98.56%	13.80	SAMN25173824
1219-PR-SAJL-008	Príncipe	SAJL	Sao Joao Largo	Pague	2019	1.64235	7.431867	28153082	27893703	99.08%	12.64	SAMN25173825
1219-PR-SAJL-029	Príncipe	SAJL	Sao Joao Largo	Pague	2019	1.64235	7.431867	22738505	22516830	99.03%	10.40	SAMN25173826
1219-PR-SAJL-030	Príncipe	SAJL	Sao Joao Largo	Pague	2019	1.64235	7.431867	27868954	27507092	98.70%	12.34	SAMN25173827
1219-PR-SCT2-002	Príncipe	SCT2	Santo Cristo	Pague	2019	1.62915	7.432333	29894758	29585850	98.97%	13.30	SAMN25173828
1219-PR-SCT2-003	Príncipe	SCT2	Santo Cristo	Pague	2019	1.62915	7.432333	28796529	28527501	99.07%	12.99	SAMN25173829
1219-PR-SCT2-007	Príncipe	SCT2	Santo Cristo	Pague	2019	1.62915	7.432333	27422293	27171066	99.08%	12.06	SAMN25173830
1219-PR-SCT2-009	Príncipe	SCT2	Santo Cristo	Pague	2019	1.62915	7.432333	39030542	38598432	98.89%	16.50	SAMN25173831
1219-PR-SJM-003	Príncipe	SJM	Sao Joaquim	Pague	2019	1.6205	7.377083	30605549	27313722	89.24%	12.14	SAMN25173832
1219-PR-SJM-006	Príncipe	SJM	Sao Joaquim	Pague	2019	1.6205	7.377083	36139913	35752201	98.93%	15.15	SAMN25173833
1219-PR-SJM-016	Príncipe	SJM	Sao Joaquim	Pague	2019	1.6205	7.377083	28006793	27728524	99.01%	12.61	SAMN25173834
1219-PR-SJM-017	Príncipe	SJM	Sao Joaquim	Pague	2019	1.6205	7.377083	32365271	32040924	99.00%	14.45	SAMN25173835
1219-PR-SJO-003	Príncipe	SJO	Sao Jose	Pague	2019	1.6358	7.398333	31816529	31505194	99.02%	13.43	SAMN25173836
1219-PR-SJO-004	Príncipe	SJO	Sao Jose	Pague	2019	1.6358	7.398333	33275911	32936240	98.98%	14.13	SAMN25173837
1219-PR-SJO-011	Príncipe	SJO	Sao Jose	Pague	2019	1.6358	7.398333	31102170	30832034	99.13%	14.02	SAMN25173838
1219-PR-SJO-016	Príncipe	SJO	Sao Jose	Pague	2019	1.6358	7.398333	26866801	26444207	98.43%	11.77	SAMN25173839
1219-PR-SRA-003	Príncipe	SRA	Santa Rita	Pague	2019	1.676783	7.412183	30668715	30410949	99.16%	13.64	SAMN25173840
1219-PR-SRA-004	Príncipe	SRA	Santa Rita	Pague	2019	1.676783	7.412183	23546002	23372918	99.26%	10.61	SAMN25173841
1219-PR-SRA-009	Príncipe	SRA	Santa Rita	Pague	2019	1.676783	7.412183	27574863	27310130	99.04%	12.59	SAMN25173842
1219-PR-SRA-011	Príncipe	SRA	Santa Rita	Pague	2019	1.676783	7.412183	35727156	35195164	98.51%	15.10	SAMN25173843
1219-PR-SUD-001	Príncipe	SUD	Sundy	Pague	2019	1.655117	7.398467	26752134	26535335	99.19%	11.83	SAMN25173844
1219-PR-SUR-005	Príncipe	SUR	Sundy	Pague	2019	1.670283	7.384317	27327320	27084231	99.11%	12.09	SAMN25173845
1219-PR-SUR-006	Príncipe	SUR	Sundy	Pague	2019	1.670283	7.384317	26636336	26390931	99.08%	11.90	SAMN25173846
1219-PR-SUR-009	Príncipe	SUR	Sundy	Pague	2019	1.670283	7.384317	24102439	23778735	98.66%	10.81	SAMN25173847
1219-PR-SUV-001	Príncipe	SUV	Sundy	Pague	2019	1.6649	7.384017	29953228	29659889	99.02%	13.30	SAMN25173848
1219-PR-SUV-002	Príncipe	SUV	Sundy	Pague	2019	1.6649	7.384017	30826597	30519672	99.00%	13.68	SAMN25173849
1219-PR-SUV-003	Príncipe	SUV	Sundy	Pague	2019	1.6649	7.384017	30010682	29727175	99.06%	13.37	SAMN25173850
1219-PR-SUV-004	Príncipe	SUV	Sundy	Pague	2019	1.6649	7.384017	30466218	30183347	99.07%	13.62	SAMN25173851
1219-PR-TVH-001	Príncipe	TVH	Terreiro Velho	Pague	2019	1.611917	7.421267	32415483	32136049	99.14%	13.80	SAMN25173852
1219-PR-TVH-011	Príncipe	TVH	Terreiro Velho	Pague	2019	1.611917	7.421267	25091290	24866424	99.10%	10.99	SAMN25173853
1219-PR-TVH-013	Príncipe	TVH	Terreiro Velho	Pague	2019	1.611917	7.421267	34084991	33683099	98.82%	14.56	SAMN25173854

Supplementary Table S2 - Pairwise FST between collection site in São Tomé Island. Three-letter code was used for every site.

Average Fst	Site Code	FED3	MIC	MRP	BOE	CON	CDM2	SMA	PGA	ALM	CRU	DIV	PAN	MOC	TRD	AZE
0.0139	**FED3**															
0.0118	**MIC**	-0.0089														
0.0084	**MRP**	0.009	0.0026													
0.0036	**BOE**	-0.0186	-0.0113	-0.0079												
0.0173	**CON**	0.0164	-0.0036	-0.0015	0.0038											
0.0103	**CDM2**	0.0114	0.01	-0.0006	0.015	-0.0152										
0.0021	**SMA**	-0.0014	-0.012	-0.0048	0.0018	-0.0097	-0.0041									
0.0023	**PGA**	-0.0206	0.0023	0.0038	-0.0223	0.0093	-0.0031	-0.0168								
0.0221	**ALM**	0.0426	0.0249	0.008	0.0316	0.0097	-0.0218	0.0074	0.0224							
0.0074	**CRU**	0.0151	0.0038	0.0038	0.0008	0.0056	-0.0039	0.0018	0.0009	-0.001						
0.0308	**DIV**	0.0538	0.0349	0.0249	0.0363	0.024	-0.0121	0.0096	0.0342	-0.022	0.0132					
-0.0012	**PAN**	0.0141	-0.0064	-0.0153	-0.0077	-0.0102	-0.0145	-0.0162	-0.0146	-0.0176	-0.0233	-0.0037				
0.0283	**MOC**	0.0557	0.0293	0.0082	0.0341	0.0193	-0.0109	0.005	0.0317	-0.0034	-0.0063	-0.0032	-0.0156			
0.0022	**TRD**	0.0229	0.0065	-0.0116	0.007	0.0114	-0.0018	-0.0067	-0.0118	-0.0051	-0.0201	0.018	-0.0281	-0.0062		
0.0342	**AZE**	0.0425	0.0326	0.0229	0.0285	0.0269	0.0307	0.0207	0.021	0.0553	0.0237	0.058	0.0058	0.0324	0.0094	
-0.0023	**MEA**	-0.0137	-0.0152	-0.0171	-0.017	-0.0006	-0.0024	-0.0113	-0.0196	0.0108	-0.0062	0.0163	-0.0238	0.0156	-0.0126	0.0114
0.0119	**NDA**	0.0306	0.0197	0.0027	-0.0006	0.0238	0.0262	-0.0097	0.0037	0.0221	0.0117	0.0401	-0.0037	0.0444	-0.0044	0.0378
0.0039	**RBA**	0.0014	0.0152	-0.0068	-0.0033	0.0108	-0.0031	-0.0025	-0.0076	-0.0007	-0.0139	0.0053	-0.0194	0.0203	-0.022	0.033
0.0039	**SNA**	0.0021	-0.0046	-0.0013	-0.0151	-0.0157	0.0115	-0.0165	0.0021	0.023	-0.0172	0.0209	-0.012	0.0145	-0.0069	0.0144
-0.0028	**UBU**	-0.0045	-0.015	-0.0118	-0.0073	-0.0201	-0.0233	-0.0286	-0.0106	-0.0075	-0.0089	-0.0006	-0.0284	0.0019	-0.0173	0.0121
0.0053	**CDF**	-0.0252	-0.0032	0.0019	-0.0057	0.0068	-0.0097	-0.0144	-0.024	0.0148	0.0123	0.03	-0.0017	0.0241	0.0048	0.0321
0.0091	**MTC**	-0.0084	0.0111	0.0106	-0.0007	0.0232	0.0072	-0.002	-0.018	0.029	0.0224	0.0236	0.0064	0.0382	0.0035	0.058
0.0087	**PIN**	0.0027	0.001	0.008	-0.0116	0.0189	0.0075	-0.0124	-0.011	0.0207	0.0134	0.0204	-0.0093	0.0328	-0.0024	0.0342
0.0081	**NEV**	0.0259	0.0106	0.0044	0.0027	0.0198	0.0129	0.0031	0.0083	0.0143	-0.0113	0.0394	-0.0096	0.011	-0.0296	0.0135
0.0176	**RLE**	-0.0055	0.0242	0.0191	-0.0122	0.0293	0.0148	0.0025	-0.0176	0.0399	0.0311	0.0474	0.0218	0.0548	0.0259	0.0451
0.0136	**STC2**	0.0015	0.021	0.0147	-0.0026	0.0526	0.0337	0.0159	-0.0054	0.0443	0.0146	0.0556	0.0165	0.0572	-0.0025	0.0442
0.0163	**CLO**	0.0117	0.018	0.0225	-0.0071	0.0427	0.0359	0.0144	0.0099	0.0568	0.0257	0.0606	0.0196	0.0582	0.0123	0.0319
0.0535	**MAL**	0.0684	0.0668	0.0605	0.0494	0.0833	0.0921	0.0605	0.0529	0.1038	0.0434	0.1164	0.0678	0.1036	0.0452	0.0852
0.0092	**MOM**	0.0138	0.014	0.0126	0.0041	0.0148	0.0094	0.0106	0.0119	0.0234	0.0148	0.0341	0.0046	0.0426	0.0068	0.039
0.0274	**POA1**	0.031	0.0467	0.0427	0.0151	0.0561	0.0584	0.0327	0.0196	0.0602	0.0211	0.0685	0.0281	0.0795	0.0221	0.0453
0.0522	**ANG**	0.0508	0.0516	0.0529	0.0303	0.0926	0.0708	0.0552	0.0383	0.0783	0.0521	0.0859	0.0504	0.0895	0.0515	0.0874
0.0073	**ATP**	0.0143	-0.0021	0.0048	0.0009	0.0126	-0.0009	-0.0071	0.0018	0.02	0.0117	0.0251	0.0086	0.0187	0.0097	0.024

Site Code	MEA	NDA	RBA	SNA	UBU	CDF	MTC	PIN	NEV	RLE	STC2	CLO	MAL	MOM	POA1	ANG
**NDA**	0.0059															
**RBA**	-0.0219	0.0131														
**SNA**	-0.0086	0.0077	-0.0152													
**UBU**	-0.0144	-0.003	-0.0133	-0.0161												
**CDF**	-0.0233	0.0112	0.0066	0.0116	-0.0063											
**MTC**	-0.0148	-0.0094	-0.0008	0.0056	0.001	-0.0191										
**PIN**	-0.0045	0.0024	-0.0106	0.01	-0.0122	-0.0091	-0.0126									
**NEV**	-0.0087	-0.0066	-0.0066	-0.0079	-0.0121	0.0248	0.0136	0.0186								
**RLE**	0.0182	0.0145	0.0208	0.014	-0.0017	-0.0093	-0.0047	0.0043	0.0267							
**STC2**	0.0049	0.0105	-0.0066	0.0083	0.0184	0.004	-0.0003	0.0139	-0.0072	0.0007						
**CLO**	-0.0006	0.0186	0.0178	0.0032	0.0115	0.007	0.0131	0.0053	0.005	0.0109	-0.0054					
**MAL**	0.0428	0.0274	0.045	0.033	0.0654	0.0584	0.0489	0.0733	0.0233	0.0577	-0.0019	0.0049				
**MOM**	0.0016	-0.0016	0.0064	0.0033	-0.004	-0.0137	-0.0042	-0.0063	0.0102	0.0069	-0.0025	-0.0088	0.0035			
**POA1**	0.0114	0.0047	0.0289	0.0231	0.0289	0.0285	0.0277	0.0297	0.0179	0.0193	-0.0086	-0.0164	-0.0196	-0.0014		
**ANG**	0.0327	0.0302	0.0514	0.0566	0.0496	0.0424	0.0338	0.0537	0.0427	0.0434	0.0189	0.0301	0.0655	0.0486	0.0304	
**ATP**	-0.0071	-0.0011	-0.0007	-0.0072	-0.0098	0.007	-0.0007	0.0018	0.001	0.0027	0.0127	-0.0031	0.0323	-0.0092	0.0164	0.0507

Supplementary Table S3 - Pairwise FST between collection site in Príncipe Island. Three-letter code was used for every site.

Average Fst	Site code	PSEC	AIR	AJT	AZT	BLV	BOM	GAS	NVE2	PAC	PAD	PBA	PBT	PBU	PCA	PDS	PIA	PIH	PLAP	POR	PSE	RAD	RBF2	REZ	RFO	SAJ	SAJL	SCT2	SJM	SJO	SRA	SUV
0.0042	**PSEC**																															
-0.0049	**AIR**	-0.0173																														
-0.0102	**AJT**	-0.0206	-0.0203																													
0.0024	**AZT**	-0.0026	-0.0328	-0.0016																												
0.0003	**BLV**	-0.0285	-0.0248	-0.0361	-0.0042																											
0.0319	**BOM**	0.0327	0.0206	0.0133	0.0263	0.034																										
0.0058	**GAS**	-0.0135	0.0047	-0.0054	-0.002	-0.0141	0.0286																									
0.0129	**NVE2**	0.0224	0.0169	-0.0022	0.0243	0.0213	0.0353	0.0072																								
0.0079	**PAC**	0.0026	0.0218	-0.0116	0.0097	0.008	0.0363	-0.0281	0.0151																							
0.0259	**PAD**	0.0061	0.0016	-0.008	0.0184	0.0045	0.034	0.0279	0.056	0.0671																						
0.0240	**PBA**	0.0063	-0.0119	0.0117	0.0185	0.0027	0.0382	0.0364	0.0309	0.0367	0.0525																					
-0.0072	**PBT**	-0.0003	-0.0164	-0.0149	-0.0177	-0.0043	0.0208	-0.0057	0.0081	-0.0115	0.0306	0.0046																				
-0.0127	**PBU**	-0.0191	-0.0222	-0.0532	-0.0163	-0.0297	0.0191	-0.0097	-0.0079	-0.0079	0.0063	-0.0049	-0.0295																			
0.0169	**PCA**	0.0067	-0.0088	0.0073	-0.0085	0.0005	0.0549	0.0428	0.0294	0.0375	0.037	-0.0098	0.0049	-0.0054																		
0.0039	**PDS**	0.0107	0.0027	-0.0328	0.013	0.0034	0.0461	0.0047	0.01	-0.0078	0.0007	0.0448	0.007	-0.0196	0.0208																	
0.0074	**PIA**	0.0104	-0.0116	-0.0006	-0.0051	0.0095	0.0354	0.03	0.022	0.0059	0.0417	-0.0025	-0.0274	-0.0108	-0.0137	0.0154																
0.0162	**PIH**	0.0074	0.0005	0.0017	0.0131	-0.0046	0.0556	-0.0147	0.0169	-0.0284	0.0514	0.0399	0.0121	0.01	0.0418	-0.0007	0.0487															
0.0094	**PLAP**	0.0222	0.0105	0.0019	0.0061	0.0161	0.0447	-0.0052	0.0196	0.0224	0.0218	0.0456	-0.0367	-0.0276	0.0171	-0.0004	0.0091	0.0237														
0.0044	**POR**	0.0193	-0.0181	0.0034	-0.0296	0.0045	0.029	-0.0091	-0.0105	0.0156	0.0252	0.0384	-0.0115	-0.0163	0.0135	-0.0086	0.001	0.0389	-0.0071													
0.0105	**PSE**	-0.0156	-0.0017	-0.0044	-0.0184	0.0051	0.034	-0.0221	0.0169	-0.0235	0.0542	0.0387	0.0139	-0.0012	0.034	0.0162	0.0107	0.0035	0.0484	0.0147												
0.0112	**RAD**	0.0332	0.0106	-0.0253	0.0135	0.0296	0.0115	0.0192	-0.0082	0.0068	0.0422	0.0473	-0.0057	-0.0113	0.033	-0.0011	0.0071	0.0219	-0.0059	-0.0007	0.0263											
0.0094	**RBF2**	-0.0012	0.0022	-0.0177	0.0131	0.0094	0.0318	0.008	0.0107	0.0146	-0.0052	0.0177	0.0011	-0.0065	0.0232	-0.0085	0.0186	0.0148	-0.0054	0.0169	0.0257	-0.0048										
0.0284	**REZ**	0.0563	0.0317	0.023	0.0397	0.043	0.0673	0.0411	0.0003	0.002	0.0604	0.0623	-0.0062	0.0238	0.0513	0.0171	-0.0005	0.0223	0.0274	0.0038	0.0342	0.0346	0.0522									
-0.0144	**RFO**	-0.0164	-0.0203	-0.0263	-0.0225	-0.0098	0.0148	-0.0262	-0.0235	-0.0218	0.0339	-0.0086	-0.0516	-0.0305	0.0056	-0.0052	-0.022	-0.0242	-0.0126	-0.0083	-0.0254	-0.015	-0.0185	-0.0122								
-0.0028	**SAJ**	0.0107	-0.0225	-0.0061	-0.0205	-0.0166	0.0232	-0.0038	0.0118	0.0119	0.025	0.0211	-0.042	-0.037	0.0085	-0.0025	-0.0032	0.019	-0.0281	-0.0284	0.0126	-0.0011	0.0173	0.0078	-0.0234							
-0.0002	**SAJL**	0.0002	0.0038	-0.0266	0.0215	-0.0093	0.037	0	0.0059	-0.0077	0.0119	0.0298	-0.0159	-0.028	0.0056	-0.0298	0.0008	0.0029	-0.004	0.0099	0.0157	0.0137	-0.0171	0.0089	-0.0195	-0.0141						
0.0186	**SCT2**	0.0304	-0.0064	0.0018	0.0094	0.0184	0.0409	0.017	0.024	0.0376	0.0179	0.0427	-0.0023	0.008	0.0287	0.0115	0.0195	0.0354	0.0099	-0.0228	0.0419	0.004	0.0134	0.0275	0.0074	-0.012	0.0069					
0.0037	**SJM**	0.0091	-0.0017	-0.015	-0.0038	0.0025	0.0465	-0.0005	0.0144	-0.003	0.0299	0.0391	-0.0272	-0.0369	0.0366	-0.0134	-0.0086	0.0287	-0.019	0.0014	-0.0088	0.0213	0.0105	0.0114	-0.0256	-0.0173	-0.019	0.0416				
-0.0081	**SJO**	-0.0214	-0.0191	-0.0184	-0.0169	-0.0128	0.0099	-0.0236	-0.0345	-0.0098	0.0144	0.0084	-0.0026	-0.0151	-0.0165	-0.0046	0.0119	-0.0172	0.0078	-0.0141	-0.0227	-0.0047	0.015	0.0171	-0.0349	-0.0161	0.0024	0.0125	0.0036			
0.0094	**SRA**	-0.0194	0.0111	-0.0249	0.0293	-0.0207	0.0387	-0.0029	0.0025	0.0003	0.0253	0.0491	0.003	-0.0113	0.0328	0.0032	0.0265	0.0011	0.0162	0.033	0.0021	0.0118	0.0182	0.0349	-0.0124	0.0071	-0.0132	0.0395	0.0056	-0.0226		
0.0153	**SUV**	0	-0.0152	-0.0161	0.0088	-0.0118	0.0052	0.0538	0.028	0.0409	0.0026	-0.0032	0.0011	-0.0112	0.0046	0.0204	0.021	0.0411	0.0423	0.038	0.0324	0.0215	0.0111	0.0655	0.0089	0.0214	0.0097	0.0371	0.0231	-0.0031	0.0009	
0.0133	**TVH**	0.0179	-0.0203	0.0091	0.0119	0.0238	0.0242	0.0463	0.0375	0.0117	0.0162	0.0203	-0.0013	0.0079	0.0086	0.0068	-0.0113	0.039	0.0318	0.014	-0.0133	0.0222	0.0315	0.0332	0.0007	0.0093	0.0121	0.0364	-0.0109	-0.0234	0.0254	-0.0053

Supplementary Figure 1. Genome scan of mean F_{ST} values between *Anopheles coluzzii* populations from São Tomé and Príncipe. Heterochromatic regions were removed from the analysis.

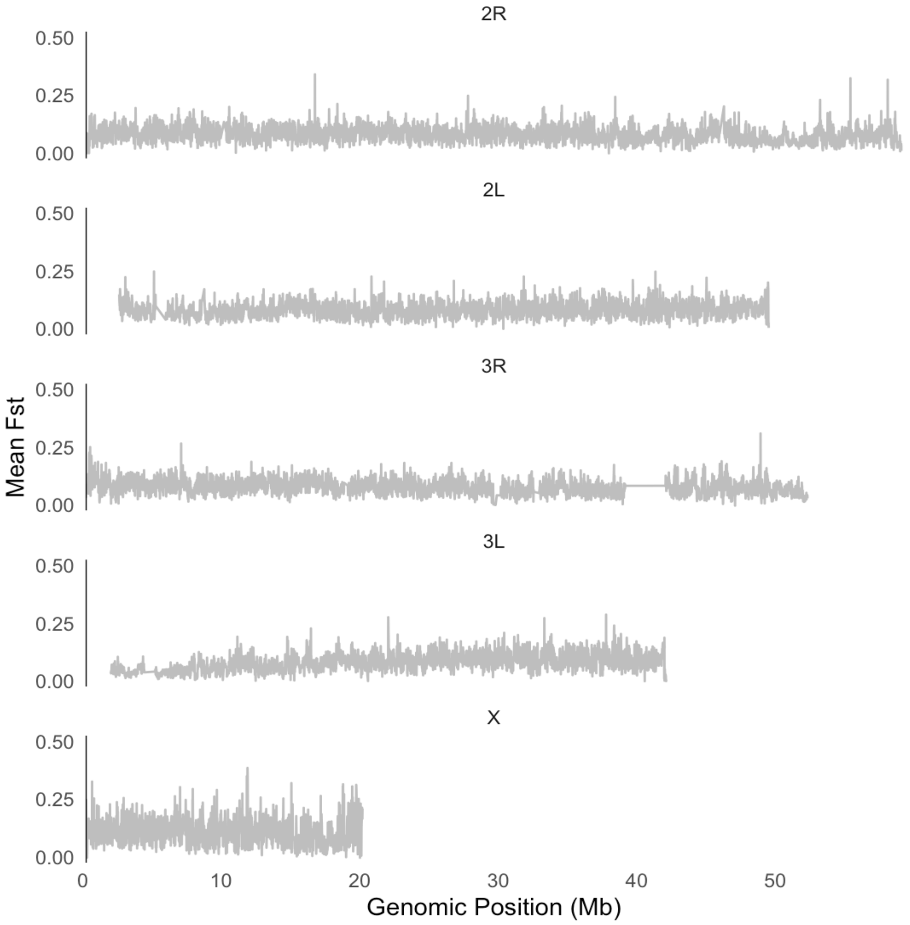

Supplementary Figure 2. Scree plot for principal component analysis. Two scree plots corresponding to the PCAs shown in Figure 4. The x-axis represents the principal components, while y-axis indicates the proportion of variance explained by each component.

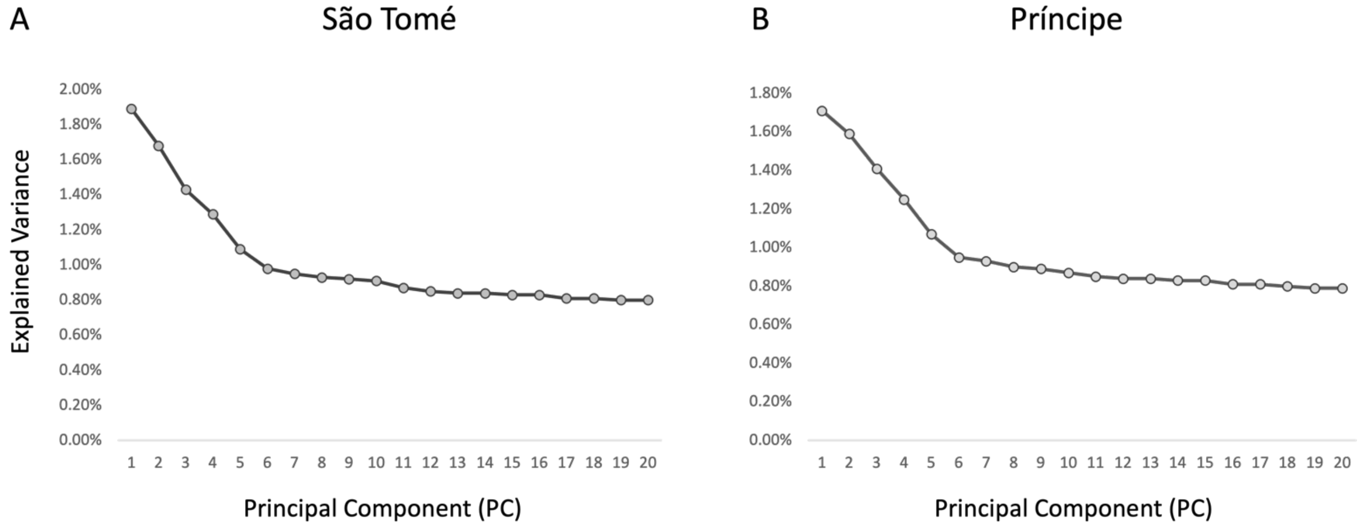

Supplementary Figure 3. Neighbor-joining tree of pairwise F_{ST}. Pairwise F_{ST} between collection sites in São Tomé (A) and Príncipe (B). Three letter code for each collection site is displayed on the maps.

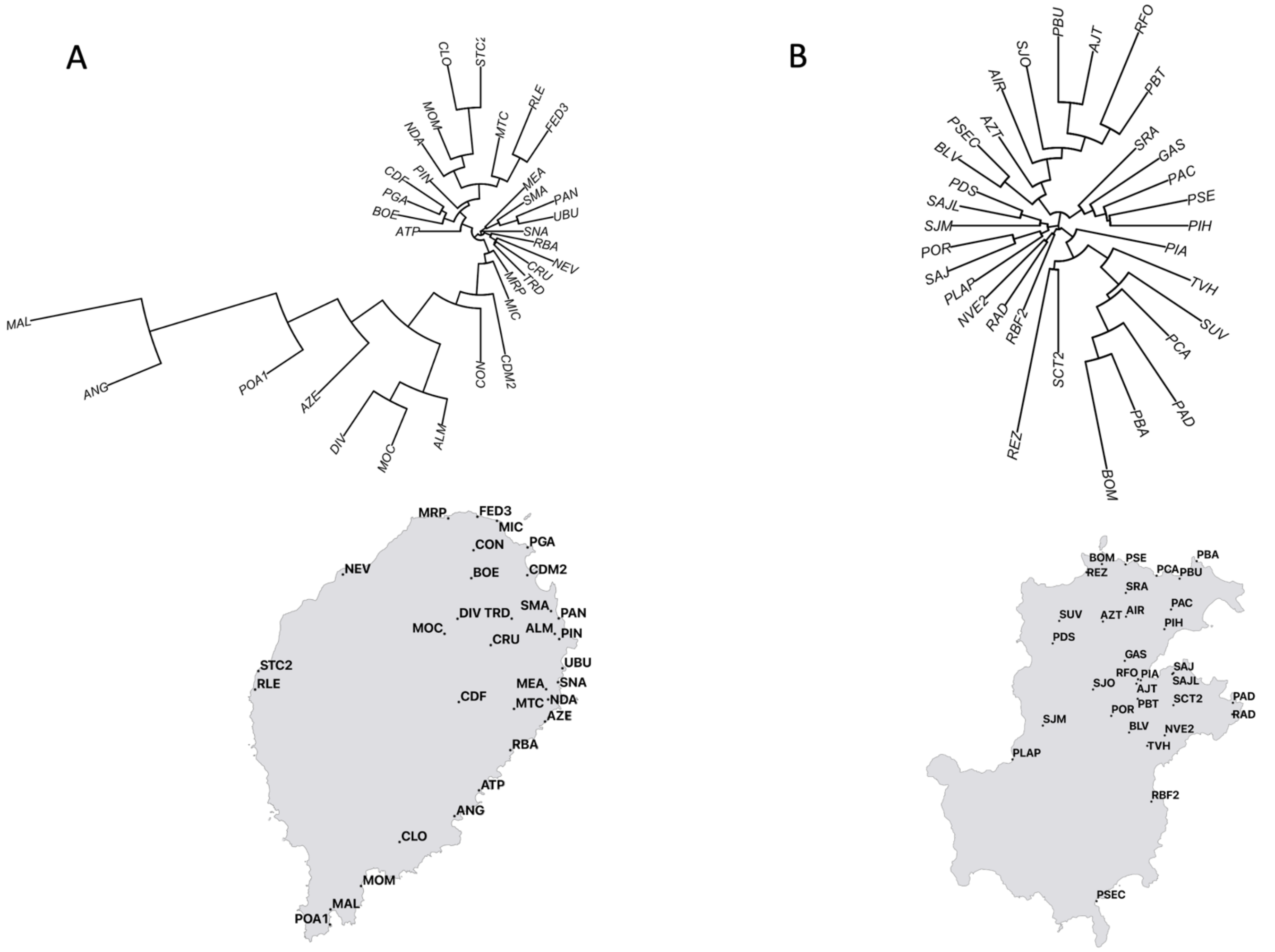

Supplementary Figure 4. Training of *disperseNN2*. Plot of training history, x-axis is the training iteration, and y-axis is mean squared error for São Tomé analysis (A) and Príncipe (B).

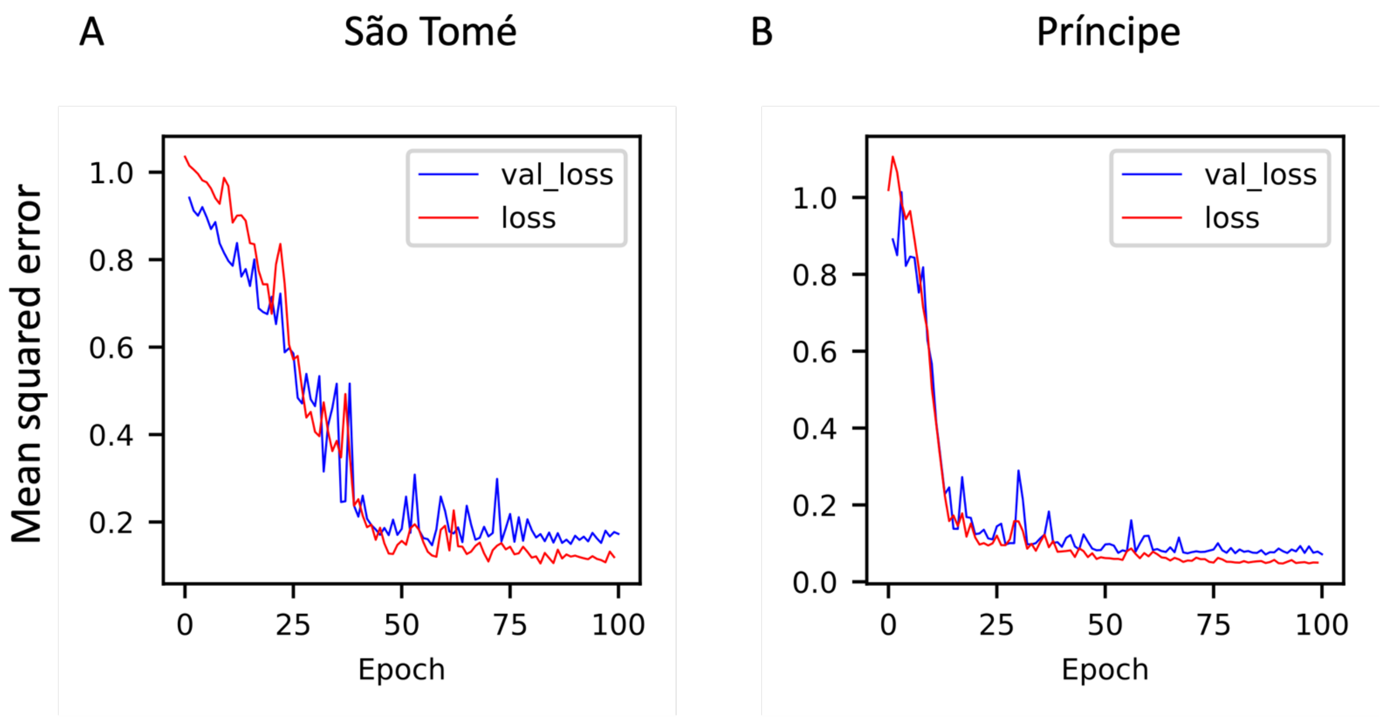

Supplementary Figure 5. Validation of *disperseNN2*. Plot of the validation of training run, true dispersal from simulation runs is on the x-axis and predicted values are on the y-axis for São Tomé (A) and Príncipe (B) analysis.

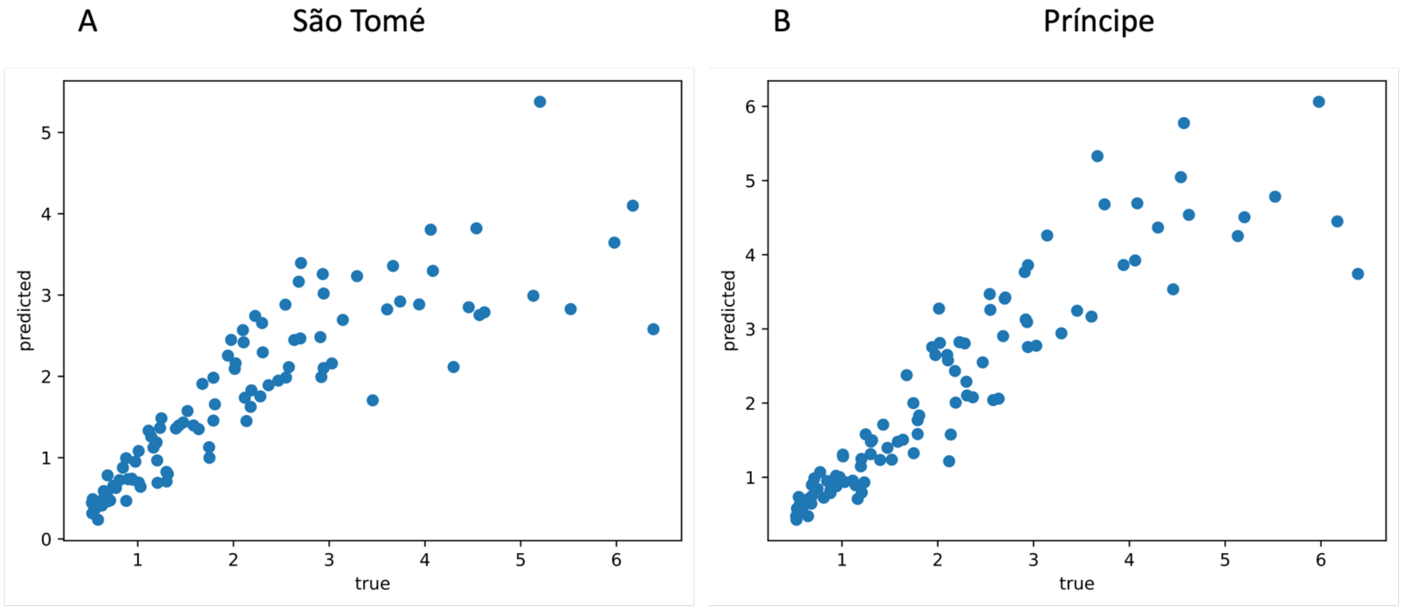

Supplement: Supplementary file 1 — Data S1. [file EVA-17-e70044-s001.pdf]
